# Kinetics of Hypohalous Acid Intermediates Governing Disinfection Byproduct Formation in Peracetic Acid-Treated Halide-Containing Waters

**DOI:** 10.1021/acs.est.5c12123

**Published:** 2026-01-08

**Authors:** Jiaqi Li, Samantha DiLoreto, Ching-Hua Huang

**Affiliations:** School of Civil and Environmental Engineering, 1372 Georgia Institute of Technology, Atlanta, Georgia 30332, United States

**Keywords:** peracetic acid, halides (bromide and iodide), hypohalous acid, halogenated disinfection byproducts, kinetic modeling

## Abstract

Peracetic acid (PAA) has emerged as an alternative disinfectant because of its lower reactivity with natural organic matter (NOM) and minimal halogenation. However, PAA may react with halides (Br^–^, I^–^) in water to form hypohalous acids, which can contribute to halogenated disinfection byproduct (DBP) formation. Meanwhile, coexisting hydrogen peroxide (H_2_O_2_) can reduce HOBr/HOI back to halides. To assess and mitigate the DBP risks of PAA in halide-containing waters, this study investigated the oxidant change and DBP formation in PAA/halide/NOM systems. A refined kinetic model accurately simulates PAA/Br^–^ and PAA/Br^–^/NOM reactions, revealing that a small fraction of NOM is highly reactive with HOBr and drives brominated DBP formation. Dibromoacetic acid dominated the identified DBPs, while total organic bromine (TOBr) analysis suggested a majority of the unidentified byproducts. In PAA/I^–^ and PAA/I^–^/NOM reactions, newly determined rate constants enabled good model and experimental data agreement. With NOM, total organic iodine (TOI) formation was the dominant iodide sink, with limited iodate formation. Triiodomethane and triiodoacetic acid were the major identified DBPs, along with more unidentified byproducts. Some unknown iodine reactive species were observed to persist in PAA/I^–^/NOM systems for hours. Importantly, applying a PAA/H_2_O_2_ molar ratio of 1:2 substantially mitigated DBP formation, decreasing known Br- or I-DBPs by 85–90% and the TOBr/TOI by 33–44%.

## Graphical abstract

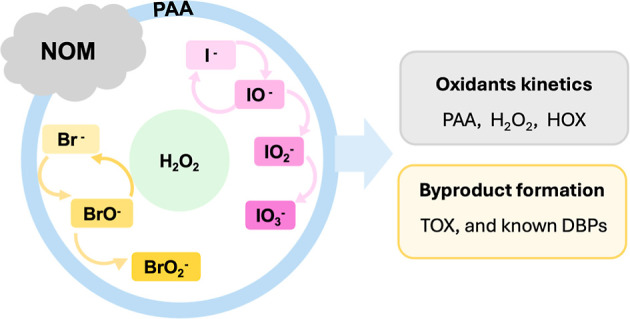

## Introduction

Peracetic acid (PAA, CH_3_C­(O)­OOH) is an organic peroxyacid widely used for disinfection in the food industry, sterilization in medical settings, catalysis in polymer synthesis, surface cleaning, and pulp bleaching.^1–3^ The commercial PAA solutions are synthesized from acetic acid (CH_3_C­(O)­OH) and hydrogen peroxide (H_2_O_2_) in the presence of catalysts, resulting in an equilibrium mixture containing PAA, H_2_O_2_, residual acetic acid, and water.^1^ In recent years, PAA has emerged as a promising alternative to chlorine for municipal wastewater disinfection due to its strong antimicrobial efficacy, chemical stability, ease of handling and storage, and compatibility with existing infrastructure.^4^ It has been extensively applied for wastewater disinfection in Europe and Canada for many years, and now in the U.S. The EPA recommends PAA as an alternative to chlorine for combined sewer overflow, wastewater, and stormwater disinfection.^5^ As a result, a growing number of full-scale wastewater treatment plants (WWTPs) in the U.S. have implemented PAA disinfection.^6^

While conventional disinfectants such as chlorine and chloramines are known to form carcinogenic halogenated disinfection byproducts (DBPs),^7,8^ PAA has been shown to form fewer DBPs under typical treatment conditions, largely attributed to PAA’s nonhalogenated nature and its selective reactivity with nucleophilic and electron-rich functional groups of organic matter.^1,4,9–11^ For example, a full-scale study across 15 WWTPs in the U.S. found that switching the disinfectant from chlorine to PAA reduced regulated trihalomethanes (THMs) and haloacetic acids (HAAs) by 95–100%. As for nitrogenous DBPs, it was found that PAA consistently yielded lower concentrations of seven nitrosamines compared to monochloramine and free chlorine.^12^

However, the presence of halide ions—chloride (Cl^–^), bromide (Br^–^), and iodide (I^–^)—can influence DBP formation during PAA disinfection for saline wastewater. Earlier studies have shown that PAA can oxidize Cl^–^, Br^–^, and I^–^ to generate reactive halogen species HOX (HOCl, HOBr, and HOI), with second-order reaction rate constants following the order of *k*_I^–^_ (389 M^–1^·s^–1^) > *k*_Br^–^_ (0.2 M^–1^·s^–1^) > *k*_Cl^–^_ (0.0021 M^–1^·s^–1^). Based on these studies,^13,14^ the important pathways in PAA/halide systems are illustrated: (1) reaction between PAA and halides forming HOX; (2) further oxidation of HOX by excess PAA, producing bromate (BrO_3_^–^) and iodate (IO_3_^–^) via bromite (BrO_2_^–^) and iodite (IO_2_^–^) intermediates—where HOI is oxidized to IO_3_^–^ more rapidly than HOBr to BrO_3_^–^; and (3) reduction of HOX back to halide ions by H_2_O_2_, forming a redox cycle that continuously regenerates reactive halogen species. Therefore, disinfection of saline wastewater using PAA may lead to the formation of brominated and iodinated DBPs. The sources of saline wastewater could be highly diverse, including (1) industrial point discharges, such as those from seafood processing, petrochemical production, tanneries, and textile industries;^15–17^ (2) the reclamation of high-salinity streams mixed with wastewater;^18^ and (3) the use of seawater for toilet flushing in many coastal cities.^19,20^ PAA is also used as a chemical disinfectant to treat ballast water on oceangoing ships.^9,13^

Previous studies have investigated the enhanced degradation of phenolic compounds during PAA oxidation in the presence of halides (bromide and iodide), with some reporting the formation of brominated and iodinated DBPs.^21–24^ Natural organic matter (NOM) in water is a major source of DBP precursors and can complicate reaction pathways by interacting with HOX. The diverse characteristics of NOM, such as molecular size, chemical composition, charge density, polarity, and hydrophobicity, can lead to the formation of a wide range of DBPs during disinfection.^25^ Some studies have assessed the formation of brominated and iodinated DBPs during PAA disinfection in the presence of halides in real water samples.^13,14^ However, no kinetic studies have examined the dynamics of PAA, H_2_O_2_, HOX, or DBP formation when NOM is present in PAA/halide systems. This knowledge gap is largely due to the analytical challenge of accurately quantifying coexisting oxidants (PAA, H_2_O_2_, and HOX) in time-resolved samples.

In this study, we first validated analytical methods for quantifying the time-dependent concentrations of three coexisting oxidants (PAA, H_2_O_2_, and HOX) in water samples. Using the validated analytical methods, various experimental measurements were obtained and utilized to refine a kinetic model for reactions in the PAA with bromide or iodide systems.^14^ Then, three NOM surrogates with different properties were selected to assess their effects on the decay of PAA and H_2_O_2_ and the formation of HOX during PAA disinfection in the presence of bromide or iodide. The NOM–HOX reactions were incorporated into the kinetic model by fitting the experimental oxidant kinetic data. The formation of brominated and iodinated DBPs, including trihalomethanes (THMs), haloacetic acids (HAAs), haloacetamides (HAMs), and haloacetonitriles (HANs), was quantified. In addition, the influence of varying H_2_O_2_ concentrations was evaluated. Based on the results, the mechanisms and implications of DBP formation in PAA-treated NOM-halide waters are discussed.

## Materials and Methods

### Chemicals and Reagents

The PAA solution (5.46 M coexisting with 1.90 M H_2_O_2_), H_2_O_2_ solution (9.98 M), and free chlorine (NaOCl) solution were purchased from Sigma-Aldrich (St. Louis, MO). The HOBr solution was freshly synthesized according to a previous method by mixing sodium hypochlorite and bromide at a molar ratio of 1:1.05 and adjusting the pH to 11 using sodium hydroxide.^26^ The HOI solution was freshly prepared by mixing sodium hypochlorite with iodide at a molar ratio of 1:1.05 with 2.5 mM borate buffer at pH 8.0.^27^ Standards of DBPs and other chemicals were purchased from different suppliers, with details provided in the Supporting Information, Text S1. Three NOM surrogates, Mississippi River reverse osmosis (RO) isolate (MS-RO), Suwannee River RO isolate (SR-RO), and Suwannee River humic acids (SR-HA), were purchased from the International Humic Substances Society (IHSS).

### Batch Kinetic Experiments

Batch experiments were conducted to investigate the reaction mechanisms in PAA/halide mixtures with or without the presence of NOM, and the decay of the oxidants was monitored over time. Unless specified otherwise, reaction conditions were [PAA]_0_ = 100–200 μM, [H_2_O_2_]_0_ = 40–400 μM, [Br^–^]_0_ = 1–2 mM, [I^–^]_0_ = 0.3–10 μM, [NOM] = 10–20 mg/L, and pH = 7.1. All experiments were conducted at room temperature (around 25 °C) and buffered with 10 mM phosphate at pH 7.1. The reaction solutions were magnetically stirred and measured for pH right after oxidant addition, as well as throughout the experiments.

### DBP Formation Experiments

Three different NOM surrogates (MS-RO, SR-RO, and SR-HA) were tested for the formation of DBPs during PAA disinfection in the presence of either bromide or iodide. The three NOM surrogates possess distinct characteristics: MS-RO contains a high nitrogen content, SR-RO represents a more general NOM composition, and SR-HA is enriched in a highly hydrophobic humic acid fraction. Additional details of the NOMs, including dissolved organic carbon (DOC) and dissolved organic nitrogen (DON) concentrations, are provided in Table S1. Reported ambient halide concentrations in saline wastewater ranged from 253 to 682 μM for bromide and 0.166 to 0.276 μM for iodide.^9,20,28^ For bromide experiments, a dose of 1.0 mM, comparable to the upper ambient levels, was applied. For iodide experiments, an iodide dosage of 4.0 μM was selected to enable accurate quantification of oxidants and capture low concentrations of DBPs.

Each experiment was conducted in 1.0 L of a NOM-containing solution buffered with 10 mM phosphate at pH 7.1. PAA was added first, followed by addition of the halide after measuring the initial concentrations of PAA and H_2_O_2_. This sequence was selected for experimental practicality, where halides were added within seconds after PAA addition to allow for rapid sample collection to confirm the initial PAA concentration. Aliquots of samples were withdrawn at predetermined time intervals to measure the oxidant concentration changes and DBP formation over time. For bromide experiments, DBP samples were collected at 0 min, 30 min, 1, 2, 3, 4, 5, and 24 h. For iodide experiments, sampling times were 0 min, 5 min, 15 min, 1, 2, and 4 h. The time = 0 min samples were collected before adding halides. Control samples containing only NOM and PAA were incubated for 5 h for bromide experiments and 2 h for iodide experiments to assess background DBP formation. At designated time points, THM samples (50 mL) were transferred into glass vials containing 10 mM sodium thiosulfate and 16.7 g/L phosphate buffer, while HAA samples (40 mL) were transferred into vials with 10 mM sodium thiosulfate. All samples were stored at 4 °C and analyzed the next day.

### Analytical Methods

The analytical methods for measuring oxidant concentrations are summarized in detail in Text S2. Briefly, the PAA concentration was determined using the potassium iodide-*N*,*N*′-diethyl-*p*-phenylenediamine (KI-DPD) method with a UV–visible spectrophotometer.^29^ The combined concentration of PAA and H_2_O_2_ was measured spectrophotometrically by a horseradish peroxidase-2,2′-azino-bis­(3-ethylbenzothiazo-line-6-sulfonic) acid (HRP-ABTS) method detailed in our previous study.^29^ The HOBr concentration was specifically measured using a sulfuric acid-catalyzed ABTS method.^30^ The HOI concentration was quantified indirectly by analyzing the formation of iodinated phenols (2- or 4-iodophenol) using high-performance liquid chromatography (HPLC) with an SC-C18 column. In addition, 2,6-dibromophenol was another compound used to quantify HOI by forming 2,6-dibromo-4-iodophenol. Samples were collected over time into HPLC vials prespiked with 4 mM phenol or 2,6-dibromophenol. The method detection limits (MDLs) were approximately 0.02 μM for 2-iodophenol, 0.01 μM for 4-iodophenol, and 0.01 μM for 2,6-dibromo-4-iodophenol, with retention times of 1.65 min, 1.95 min, and 7.03 min, respectively. For a mixture of PAA, H_2_O_2_, and HOX, whether the combined use of the above-mentioned methods could differentiate PAA, H_2_O_2_, and HOX was validated in this study and is shown in the Results and Discussion section and Text S2.

Iodate was quantified by ion chromatography with postcolumn UV detection using a Dionex IonPac AS24 guard column (4 × 50 mm) with the analytical column (4 × 250 mm). The mobile phase consisted of 8.0 mM Na_2_CO_3_ and 1.0 mM NaHCO_3_ at a flow rate of 1.0 mL/min. The limit of detection for iodate was 0.2 μM, with a retention time of 4.4 min.

A total of 25 brominated and iodinated DBPs were included for analysis: (1) six THMs: bromodichloromethane (BDCM), dibromochloromethane (DBCM), tribromomethane (bromoform, TBM), dibromoiodomethane (DBIM), chlorodiiodomethane (CDIM), triiodomethane (iodoform, TIM); (2) four HANs: bromoacetonitrile (BAN), bromochloroacetonitrile (BCAN), dibromoacetonitrile (DBAN), iodoacetonitrile (IAN); (3) four HAMs: bromoacetamide (BAM), bromochloroacetamide (BCAM), dibromoacetamide (DBAM), iodoacetamide (IAM); and (4) 11 HAAs: monobromoacetic acid (MBAA), monoiodoacetic acid (IAA), bromochloroacetic acid (BCAA), dibromoacetic acid (DBAA), bromodichloroacetic acid (BDCAA), chlorodibromoacetic acid (CDBAA), chloroiodoacetic acid (CIAA), bromoiodoacetic acid (BIAA), tribromoacetic acid (TBAA), diiodoacetic acid (DIAA), triiodoacetic acid (TIAA).

THMs, HANs, and HAMs were extracted by liquid–liquid extraction (LLE) with methyl *tert*-butyl ether (MTBE) based on the EPA 551 method.^31^ HAAs were extracted using LLE and acid derivatization based on the EPA 552 method.^32^ Procedural calibration standards and laboratory reagent blanks (LRB) were extracted along with samples for QA/QC. Decafluorobiphenyl was added as a surrogate standard (10 ppb) for the 551 method. 2-Bromobutanoic acid was added as a surrogate standard (10 ppb), and 1,2-dibromopropane was used as an internal standard (100 ppb) for the 552 method. The detailed extraction procedures for 551 and 552 are provided in Text S3. Following extraction, samples were analyzed using gas chromatography coupled with electron capture detection (GC-ECD, 7890A GC System, Agilent Technologies, United States) with a J&W DB-1 GC column (30 m × 0.25 mm × 0.25 mm). The GC-ECD conditions for the methods are provided in Text S3. Overall, the methods yielded excellent linearity, with detection limits (MDLs) in the range of 0.006–1.684 μg/L for DBPs based on the 551 method and 0.042–0.549 μg/L for DBPs based on the 552 method.

### Data Analysis

Kinetic modeling was performed to simulate the reaction rates by fitting the model to the experimental data of changes in concentrations of PAA, H_2_O_2_, and HOBr/BrO^–^, and HOI/IO^–^ in the presence or absence of NOM using the program Kintecus 4.55.31.^33^ The fitting accuracy was evaluated by the root-mean-square deviation method in the Kintecus software. The correspondence between model predictions and experimental observations was evaluated by the calculation of the sum of deviations between predicted and measured values (eq 1), with results shown in Table S2.^31^

1 $$Overall\;SD=\sqrt{\frac{1}{N\times M-1}\sum_{j}^{M}\sum_{i}^{N}{\;\left(C_{\exp,i,j}-C_{cal,i,j}\right)}^{2}}$$

where *N* is the number of species; *M* is the number of samples collected for each species; *C*_exp,*i*,*j*_ and *C*_cal,*i*,*j*_ are the *j* th experimental and calculated concentrations of species *i*, respectively.

## Results and Discussion

### Measuring HOX, H_2_O_2_, and PAA Concentrations in PAA-Single Halide Systems

Methods used in this study to specifically quantify the concentrations of oxidants are summarized in Table 1, with target species for each method specified. Detailed validation of these methods was conducted and is described in Text S2 and Figures S1–S4. The H_2_O_2_ concentration can be determined by subtracting the combined [PAA + HOX] concentration measured by the KI-DPD method from the total oxidant concentration obtained via the HRP-ABTS method. The HOBr concentration was found to be specifically measurable by the H_2_SO_4_–ABTS method in the presence of PAA and H_2_O_2_. The HOI concentration can be measured by the formation of iodophenols in samples collected in vials prespiked with phenol. The PAA concentration can be determined indirectly by subtracting the HOBr or HOI concentration from the total concentrations of PAA and HOX measured by the KI-DPD method. In brief, the combined use of these methods enabled the simultaneous measurement of each concentration of PAA, H_2_O_2_, and HOX in the mixture of single-halide systems accurately. Even so, we think applying these combined methods to monitor oxidant changes in real wastewater samples can be challenging because, first, the presence of coexisting halides complicates the accurate differentiation and quantification of HOI and HOBr. Second, other dissolved species, such as ammonia, nitrite, and metal ions, can further interfere. Ammonia can convert HOBr to halamines, while nitrite may also react with PAA.^32–34^ Metal ions such as Fe^2+^ and Fe^3+^ can react with PAA and H_2_O_2_, generating Fenton reactions.^34^

**Table 1:** Summary of Methods for Quantification of the Concentrations of Oxidants in a PAA/H_2_O_2_/HOX Mixture

method	measured specie(s)	validation
KI-DPD method	PAA + HOX	Figure S1 and Text S2
HRP-ABTS method	PAA + HOX + H_2_O_2_	Figure S2 and Text S2
H_2_SO_4_-ABTS method	HOBr	Figure S3 and Text S2
2- and 4-iodophenol formation	HOI + I^–^	Figure S4 and Text S2

### Oxidant Kinetics in the PAA/Br^–^ Systems

Oxidant concentrations (PAA, H_2_O_2_, and HOBr) were monitored over time in PAA–bromide mixtures and compared to predictions from a kinetic model (Table 2, R1–R3 and R8), built upon previous studies.^13,14^ The changes of the oxidant concentrations were evaluated at different H_2_O_2_ (Figure 1a,b) or bromide (Figure 1b,c) concentrations with the same PAA concentration. As shown in Figure 1, across different test conditions, HOBr was observed to form only after H_2_O_2_ depletion, due to the immediate recycling of HOBr to bromide by H_2_O_2_ (R8). At the same bromide concentration but different H_2_O_2_ levels, the PAA decay remained similar. HOBr formation was slower and reached a lower maximum concentration at the higher H_2_O_2_ level (60 μM; Figure 1a), compared to the lower H_2_O_2_ level (40 μM; Figure 1b). This is because a higher H_2_O_2_ concentration promoted the recycling of the formed HOBr back to bromide. At a fixed H_2_O_2_ concentration, increasing the bromide concentration accelerated both PAA decay and HOBr formation (Figure 1a,c); however, the maximum HOBr concentration remained similar. This is because, with 100 μM PAA and excess bromide (1–2 mM), up to 100 μM HOBr can theoretically form (R1). Meanwhile, H_2_O_2_ can reduce an equivalent concentration of HOBr back to bromide. Consequently, the difference between PAA and H_2_O_2_ concentrations can determine the maximum HOBr concentration. Finally, the experimental data of oxidant concentration changes aligned closely with predictions by the kinetic model under different H_2_O_2_ and bromide concentrations, validating the model’s reliability for the PAA/bromide systems.

**Table 2:** Principal Reactions in the PAA Oxidation of Single Halide-Containing Water^a^

	reactions	*k* (M^–1^·s^–1^) at pH = 7.1	refs
R1	CH_3_C(O)OOH + Br^–^ → OBr^–^ + AA	0.198; 0.24 ± 0.02	^8^
R2	CH_3_C(O)OOH + HOBr → BrO_2_^–^	NA (slow)	^21^
R3	CH_3_C(O)OOH + BrO_2_ → BrO_3_^–^	NA (slow)	^21^
R4	CH_3_C(O)OOH + I^–^ → IO^–^ + AA	890	this study
R5	CH_3_C(O)OOH + HOI/IO^–^ → IO_2_^–^ + AA	7.38 ± 0.64	this study
R6	CH_3_C(O)OOH + IO_2_^–^ → IO_3_^–^ + AA	1000 (assumed)	^21^
R7	CH_3_C(O)OOH + NOM → products	slow	^1^
R8	H_2_O_2_ + HOBr/OBr^–^ → Br^–^	2.36 × 10^4^	^40^
R9	H_2_O_2_ + HOI/OI^–^ → I^–^ + H_2_O	2.28 ± 0.34 × 10^4^	this study
R10	HOBr/OBr^–^ + NOM_(HOBr,1)_ → TOBr_1_	1 × 10^6^	assumed
R11	HOBr/OBr^–^ + NOM_(HOBr,2)_ → TOBr_2_	5 × 10^3^	assumed
	NOM_(HOBr,1)_ for MS-RO, SR-RO, and SR-HA are 0.77, 1.07, and 2.43 μmol/mgNOM, respectively		this study
	NOM_(HOBr, 2)_ for MS-RO, SR-RO, and SR-HA are 1.88, 2.30, and2.05 μmol/mgNOM, respectively		this study

a AA = acetic acid; TOBr = total organic bromine; MS-RO, SR-RO, and SR-HA are three NOM surrogates in this study; NOM_(HOBr,1)_ denotes the fast-reactive fraction of NOM with HOBr, whereas NOM_(HOBr,2)_ represents a relatively less reactive, yet still kinetically significant, NOM fraction with HOBr. NOM fractions slow reactive with HOBr are not included. Further details are provided in the main text.

**Figure 1:**
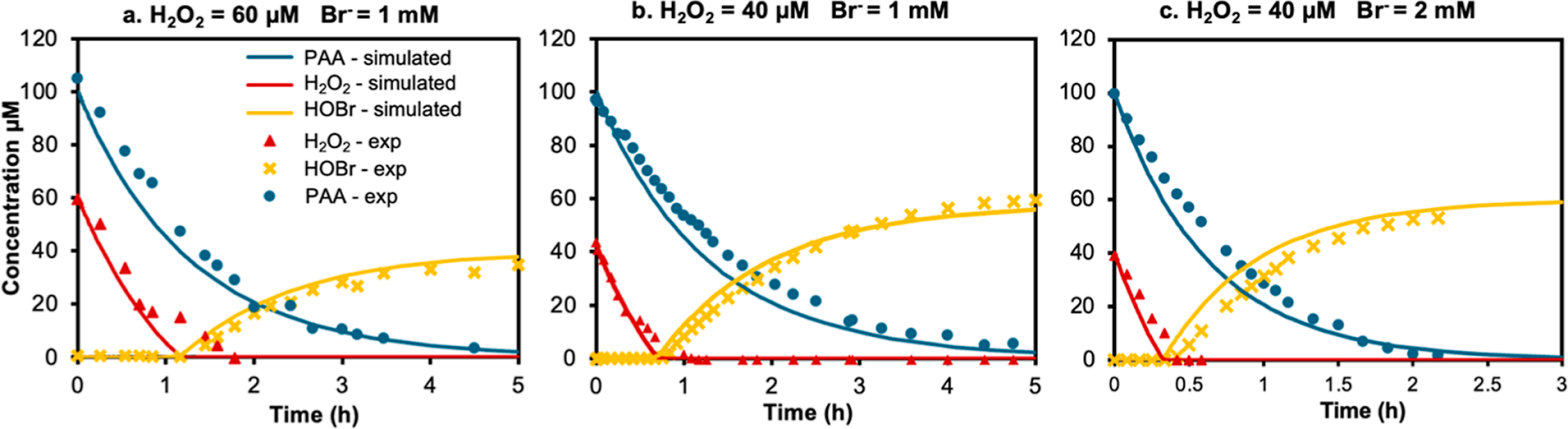
Temporal changes in the concentrations of PAA (blue), H_2_O_2_ (red), and HOBr (yellow) in the PAA/Br^–^ systems based on experimental measurements (symbols) and kinetic model simulations (solid lines). All experiments used initial concentrations of 100 μM PAA, 40 or 60 μM H_2_O_2_, 1.0 or 2.0 mM Br^–^ at pH 7.1 (10 mM phosphate buffer) and room temperature.

### Oxidant Kinetics and DBP Formation in the PAA/Br^–^/NOM Systems

With the validated kinetic model, we further investigated the oxidant kinetics and DBP formation in the copresence of PAA, bromide, and NOM. Three NOM surrogates, MS-RO, SR-RO, and SR-HA, were examined, with results shown in Figure 2. Compared to the NOM-absent condition (Figure 1a), the presence of NOM significantly slowed down the H_2_O_2_ decay (Figure 2a), which does not align with the predicted results from previous studies.^13,14^ Kinetically, the formed HOBr is consumed in two ways: (1) reacting with H_2_O_2_ and recycling back to bromide; and (2) reacting with NOM to form brominated byproducts. With 20 mg/L of different NOMs, the H_2_O_2_ decay was dramatically slowed down and nearly terminated in the presence of SR-HA. This indicates the formed HOBr primarily reacted with NOM at a reaction rate higher than that with H_2_O_2_ (*k*_(H_2_O_2_, HOBr/OBr-)_ = 2.36 × 10^4^ M^–1^·s^–1^), suggesting the previously used rate constants (e.g., *k*_(NOM, HOBr/OBr-)_ = 30–130 M^–1^·s^–1^) were significantly underestimated.^14,26,35^ In addition, when SR-RO was lowered to 10 mg/L (∼416 μM/L as carbon), there was HOBr accumulating in the system over time, indicating that only a fraction of NOM can react rapidly with HOBr in this case, with a rate higher than *k*_(H_2_O_2_, HOBr/OBr-)_ (Figure 2c).

**Figure 2:**
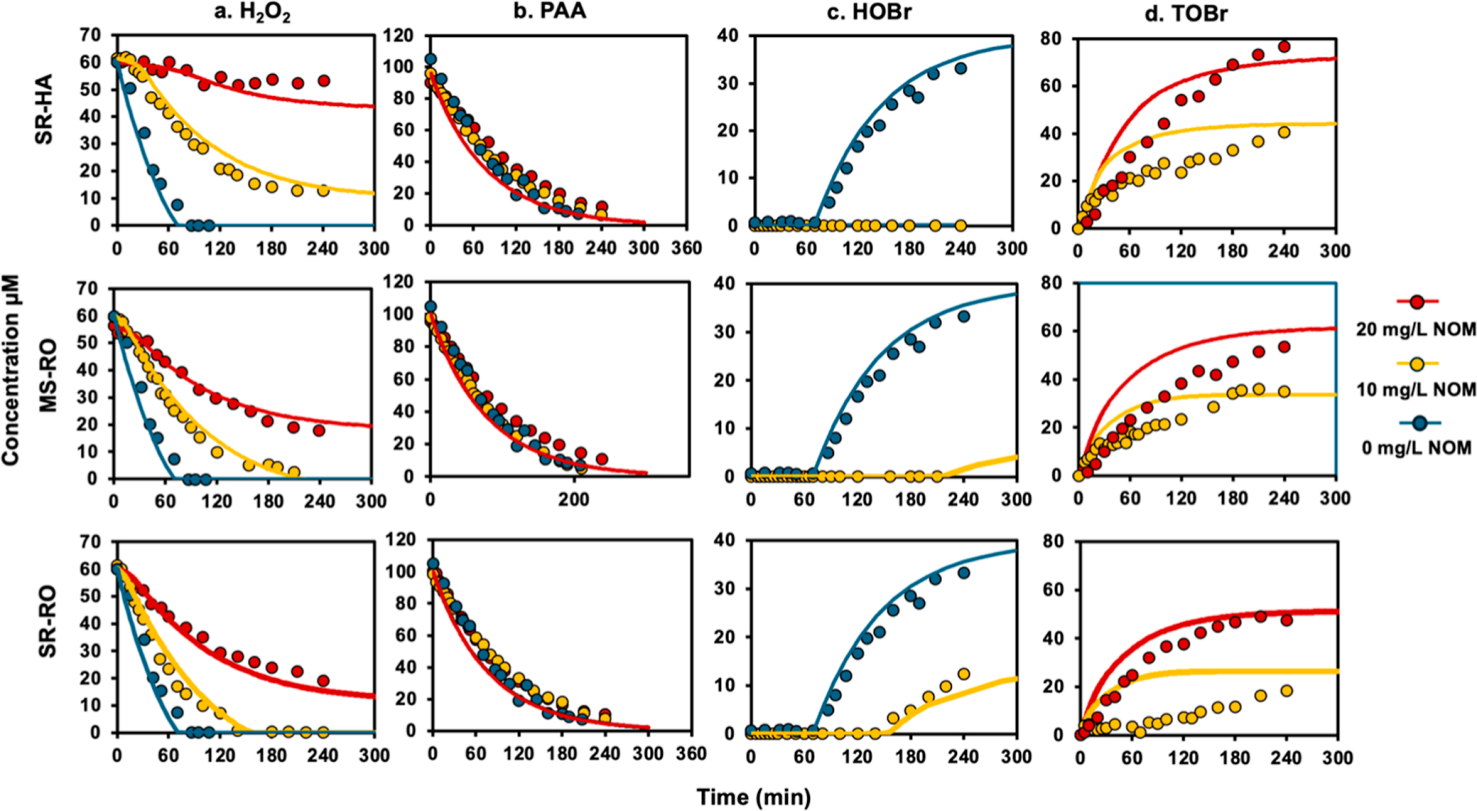
Temporal changes in the concentrations of PAA, H_2_O_2_, HOBr, and TOBr in the PAA/Br^–^/NOM systems based on experimentally measured values (symbols) and kinetic model simulations (solid lines). Three different NOM surrogates at 0 ppm (blue), 10 ppm (yellow), and 20 ppm (red) were tested. All experiments used initial concentrations of 100 μM PAA, 60 μM H_2_O_2_, and 1.0 mM Br^–^ at pH 7.1 (10 mM phosphate buffer) and room temperature.

The time-resolved measurement of the oxidant species enabled the estimation of total organic bromine (TOBr) formation based on mass balance, with detailed deduction shown below (eqs 2 and 5). The TOBr can be expressed as the difference between the decayed concentrations of PAA and H_2_O_2_ at any time point, assuming a 1:1 stoichiometric reaction between NOM and HOBr to yield TOBr as the only product. It is important to note that NOM can react with HOBr at varying stoichiometric ratios, forming mono-, di-, or multibrominated compounds. In this study, a 1:1 reaction ratio was assumed to easily estimate the TOBr concentration in μM as Br. Further, the PAA self-decay is slow with first-order rate constants ranging from 1.38 × 10^–4^ to 1.00 × 10^–3^ min^–1^, giving a half-life time of 693–5022 min,^24^ hence negligible in the presence of bromide. Control experiments showed that the PAA concentration remained stable within one hour in the presence of 5 mg of MS-RO NOM, confirming the slow reaction between PAA and NOM.

$$\frac{\Delta \left[TOBr\right]}{\Delta t}=\frac{\Delta \left[{HOBr}_{NOM}\right]}{\Delta t}=\frac{{\Delta [HOBr}_{formed}]}{\Delta t}-\frac{\Delta \left[{HOBr}_{H_{2}O_{2}}\right]}{\Delta t}-\frac{\Delta \left[{HOBr}_{accumulated\;in\;reactor}\right]}{\Delta t}$$

2 $$=\frac{\Delta {[PAA}_{consumed}]}{\Delta t}-\frac{\Delta \left[{H_{2}O_{2}}_{consumed}\right]}{\Delta t}-\frac{\Delta \left[{HOBr}_{accumulated\;in\;reactor}\right]}{\Delta t}$$

where Δ*t* = *t* – *t*_0_, with *t* being at any given time. Δ­[HOBr_formed_] represents the total HOBr formed by the reaction between PAA and Br^–^ at time *t*, as indicated by the decrease in PAA concentration (Δ­[PAA_consumed_]). Δ­[HOBr_NOM_] and Δ­[HOBr_H_2_O_2__] are the amounts of HOBr consumed by reactions with NOM and H_2_O_2_, respectively. Δ­[HOBr_accumulated in reactor_] denotes the HOBr accumulated in the reactor from *t*_0_ to *t*.

3 $$Then,\;when\;t=t_{1},\Delta {[PAA}_{consumed}]={[PAA}_{t=0}]-{[PAA}_{t=t_{1}}]$$

4 $$\Delta \left[{H_{2}O_{2}}_{consumed}\right]=\left[{H_{2}O_{2}}_{t=0}\right]-{[H_{2}O_{2}}_{t=t_{1}}]$$

5 $$\Delta \left[TOBr\right]=\left({[PAA}_{t=0}]-{[PAA}_{t=t_{1}}]\right)-\left(\left[{H_{2}O_{2}}_{t=0}\right]-{[H_{2}O_{2}}_{t=t_{1}}]\right)-\left[{HOBr}_{t=t_{1}}\right]$$

where [PAA_*t*=0_] and ${[PAA}_{t=t_{1}}]$ are the PAA concentrations in the reactor at time = 0 and *t*_1_; ${[H_{2}O_{2}}_{t=0}]$ and ${[H_{2}O_{2}}_{t=t_{1}}]$ are the H_2_O_2_ concentrations in the reactor at time = 0 and *t*_1_, respectively; and $\left[{HOBr}_{t=t_{1}}\right]$ is the HOBr concentration at time *t*_1_.

Based on eq 5, the TOBr concentrations were calculated and are shown in Figure 2d. The TOBr formation was 44.6, 47.3, and 76.7 μM for 20 mg/L MS-RO, SR-RO, and SR-HA at5 h, respectively. This indicates a significant fraction of the formed HOBr reacted with NOM, considering that only a maximum of 100 μM HOBr can theoretically form from the reaction between 100 μM PAA and 1.0 mM bromide.

The above findings demonstrated that the fast-reactive NOM dominated the reaction between NOM and HOBr in PAA/Br^–^/NOM systems, contributing to the non-negligible formation of TOBr. However, NOM is intrinsically complex and comprises components that vary in both concentration and reactivity with HOBr. To provide as much quantitative insight as possible, we attempted to estimate the amounts of fast-reactive NOM by kinetically fitting the observed oxidant decay.

Certain NOM moieties, such as hesperetin, resorcinol, and phloroglucinol, exhibited apparent second-order rate constants with HOBr ranging from 10^6^ to 10^7^ M^–1^·s^–1^ at pH 7 (Table S3).^35–37^ Thus, we assumed a reaction rate constant of 1 × 10^6^ M^–1^·s^–1^ for these fast-reactive NOM fractions (NOM_HOBr,1_). Some NOM moieties, e.g., phenol, *m*-cresol, vanillin, flavone, gallic acid, and catechol, featured moderate apparent reaction rate constants ranging from 10^4^ to 10^5^ M^–1^·s^–1^ (Table S4).^35–37^ For this relatively fast reactive fraction (NOM_HOBr,2_), an average reaction rate constant of 5 × 10^4^ M^–1^·s^–1^ was assumed. Some NOM components exhibited low reactivity toward HOBr and contributed to slower halogen consumption, with reported second-order rate constants ranging from 31 to 130 M^–1^·s^–1^ at pH 5.^35^ For these slow-reactive NOM fractions, even at a high concentration (e.g., when all spiked NOM is slow-reactive), the impact on the H_2_O_2_ decay is minimal. This is because their reaction rates with HOBr are much lower than those of the reaction between HOBr and H_2_O_2_. Thus, the slow-reactive NOM fraction was not included in the model.

First, we assumed the NOM_(HOBr, 1)_ and NOM_(HOBr,2)_ concentrations (μM/mg NOM) in each NOM surrogate are consistent at different NOM dosages. Then, kinetic fitting of the observed decay of oxidants (PAA and H_2_O_2_) under varying NOM concentrations was conducted to determine the NOM_(HOBr, 1)_ and NOM_(HOBr,2)_ concentrations. As shown in Figure 2, the model simulation can describe the observed oxidant kinetics and TOBr formation in the presence of NOM. The concentrations of fast-reactive NOM for SR-RO, M-RO, and SR-HA were determined to be 0.77, 1.07, and 2.43 μmol/mg NOM by kinetic fitting. The concentrations of relatively fast-reactive NOM for the three surrogates were similar, which were 1.88, 2.30, and 2.05 μmol/mg NOM, respectively. These levels are consistent with those reported in previous studies for real water samples, which ranged from 0.26 to 0.92 μmol/mg C, with apparent second-order rate constants between 5.4 × 10^5^ and 1.4 × 10^6^ M^–1^·s^–1^.^37^

With the presence of 1.0 mM bromide, various brominated DBPs formed gradually during PAA disinfection within the first two hours and then slowly increased in the next three hours (Figure 3). After incubation for up to 24 h, a small gradual increase in DBP formation was observed for MS-RO and SR-RO, while no further increase was detected for SR-HA, indicating that DBP formation primarily occurred within a short contact time. Across the different NOM types, MS-RO featured the highest total known DBP formation (586.5 μg/L), followed by SR-HA (507.5 μg/L) and SR-RO (487.4 μg/L) in 5 h. The total organic bromine from the detected DBPs was 5.36, 4.45, and 4.18 μM for MS-RO, SR-RO, and SR-HA, respectively, which accounted for only 12.0%, 9.4%, and 5.5% of TOBr formation in 4 h, confirming the presence of many unknown brominated DBPs or intermediates. These unknown brominated DBPs could be polar aromatic and unsaturated aliphatic Br-DBPs such as 2,4,6-tribromophenol, 3,5-dibromo-4-hydroxybenzoic acid, 2,6-dibromo-1,4-hydroquinone, and 3,3-dibromopropenoic acid, and other Br-DBPs rich in carboxyl groups.^38,39^ A recent study used Fourier transform ion cyclotron resonance mass spectrometry (FT-ICR MS) to characterize unknown Br-DBPs in artificial drinking water, revealing 441 formulas for monobrominated products and 37 formulas for dibrominated products.^38^ The abundant monobrominated products reported in that study could potentially be formed in the HOBr slow-release system of this study. Future investigation is needed on direct TOBr quantification and unknown brominated DBP identification to gain a deeper understanding of the composition of brominated DBPs.

**Figure 3:**
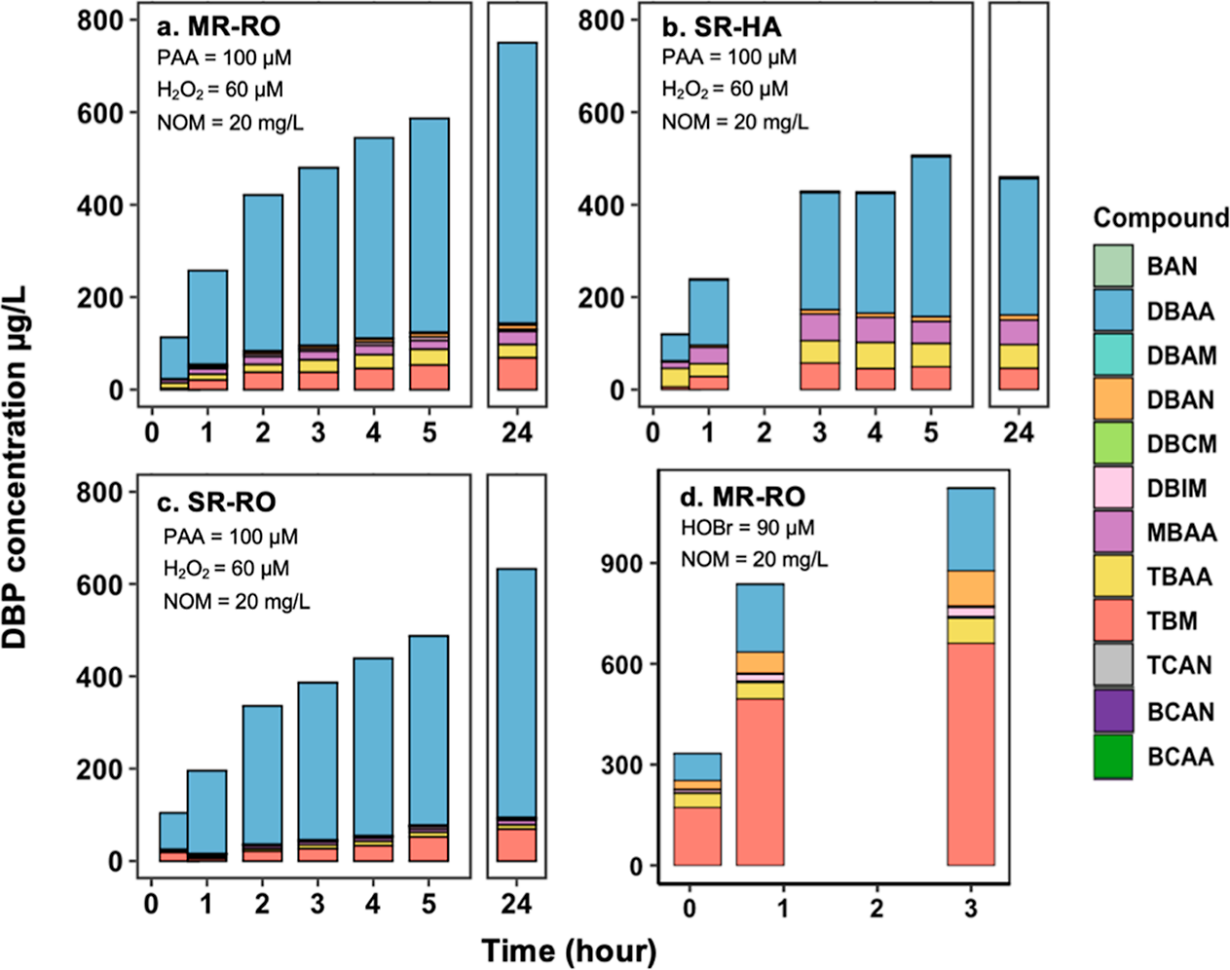
DBP formation during PAA disinfection in the presence of bromide and different NOM surrogates, including MR-RO (a), SR-HA (b), and SR-RO (c) at 20 mg/L. (d) DBP formation during direct bromination with 90 μM HOBr and 20 mg/L MR-RO. All experiments used initial concentrations of 100 μM PAA, 60 μM H_2_O_2_, and 1 mM Br^–^ at pH 7.1 (10 mM phosphate buffer) and room temperature.

For all the NOMs, dibromoacetic acid (DBAA) was the most abundant DBP, at 606.65 μg/L for MS-RO, 537.89 μg/L for SR-HA, and 295.07 μg/L for SR-RO after 24 h, contributing to 76.5%, 81.6%, and 57.6% of the calculated total organic bromine from all the known DBPs on a molar average. This is consistent with previous studies on PAA disinfection with halides, reporting that DBAA is one of the dominant byproducts.^9,13,21^ Besides DBAA, other DBPs, including TBM, MBAA, and TBAA, were also consistently detected across different NOM types, with SR-HA formed primarily brominated acetic acids and TBM. The MS-RO, featuring a higher nitrogen content (Table S1), showed the highest formation of nitrogenous DBPs, including DBAM, DBAN, and BAN. Based on the formation of TOBr and known DBPs, it is highly recommended to keep the PAA disinfection time within one hour, which can effectively limit the brominated DBP formation. However, without quenching reagents to remove PAA, the DBP can form continuously.

Interestingly, some studies have reported TBM to be the most abundant DBP during bromination.^40^ For comparison, MS-RO was used to test DBP formation during direct bromination. An initial HOBr concentration of 90 μM was used, which is comparable to the maximum level of HOBr formation in PAA–bromide experiments. After mixing 90 μM HOBr with 20 mg/L MS-RO, HOBr decayed instantaneously to 66.5 μM, followed by a fast decay to 51.6 μM within 2 min, and finally reduced to 1.92 μM in 3 h (Figure S5). Unlike the PAA/Br^–^/NOM system, TBM was the most abundant DBP with a concentration of 661.30 μg/L in direct bromination, followed by DBAA (245.48 μg/L) and DBAN (104.18 μg/L). This is consistent with previous studies that reported TBM as the dominant DBP during bromination (Figure 3d).^9,13,21,41^ The known DBPs contributed to 16.1% of TOBr in direct bromination, which is higher than that in the PAA/Br^–^/NOM systems.

We hypothesize that the high yield of DBAA and unknown brominated byproducts in the PAA/Br^–^/NOM systems is due to the limited availability of HOBr. The slow release of HOBr from the reaction between PAA and bromide and the competition between H_2_O_2_ and NOM for HOBr render HOBr only available to the most reactive precursors, e.g., DBAA precursors, and tend to form mono- or dihalogenated DBPs. For example, aliphatic β-keto acid compounds, 3-oxobutanedioic and 3-oxopentanedioic acids, are known DXAA precursors. Also, aliphatic β-dicarbonyl acids can lead to significant THM and/or DXAA formation, while the TCAA and TBAA yields were low.^42^ These aliphatic acid compounds may be the dominant precursors that are quickly reactive with HOBr in the PAA/Br^–^/NOM systems. This could also explain why the SR-HA had the lowest DBAA formation. Compared to the other two NOM isolates, SR-HA contains fewer aliphatic acid components.

### Oxidant Kinetics in the PAA/I^–^ Systems

In PAA–iodide systems (200 μM PAA with 0.3 to 10 μM iodide), concentrations of PAA, H_2_O_2_, and iodophenol formation were measured over time (Figure 4). It should be noted that the measured iodophenols represent the combined concentration of [HOI + I^–^] at each time point because HOI and iodide are involved in a cycling process when both PAA and H_2_O_2_ are present. The recycling of HOI back to iodide ceases to occur when H_2_O_2_ is depleted. Therefore, the iodophenols’ concentration measured after the complete depletion of H_2_O_2_ indicates the HOI concentration. In the absence of NOM, three primary iodinated species are present, including HOI, I^–^, and iodate. Because iodophenols’ formation reflects the combined contributions of HOI and I^–^, the iodate concentration was determined by subtracting the iodophenols’ concentration from the initial iodide concentration. As Figure 4 shows, when the initial iodide concentration was increased, the decay of PAA was accelerated, accompanied by a more rapid formation of HOI. Consequently, the depletion of H_2_O_2_ was faster, rendering a higher maximum HOI concentration that approached the initial iodide level. Since PAA can oxidize HOI further into iodate, a higher iodate formation was also observed with a higher initial iodide concentration.

**Figure 4:**
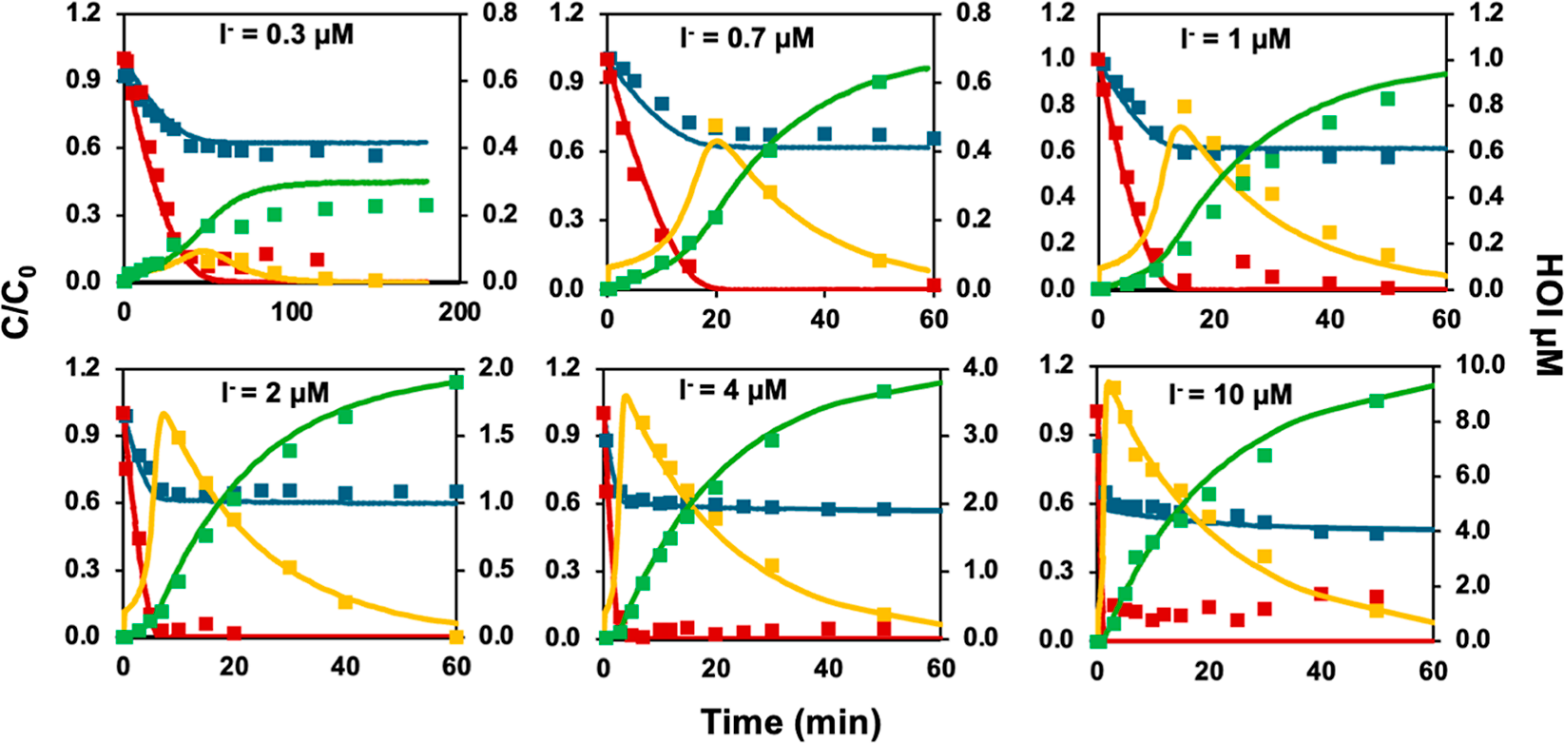
Temporal changes in the concentrations of PAA (blue), H_2_O_2_ (red), HOI (yellow), and iodate (green) in the PAA/I^–^ systems based on experimental measurements (symbols) and kinetic model simulations (solid lines). All experiments used initial concentrations of 200 μM PAA, 70 μM H_2_O_2_, and 0.3–10 μM iodide at pH 7.1 (10 mM phosphate buffer) and room temperature.

We attempted to fit the experimental results into the kinetic model previously developed (Table 2, R4–R6 and R9).^14^ At a higher iodide concentration (4 μM), experimental data matched the simulations well (Figure S6); however, differences were noted with decreasing iodide concentrations (0.3 and 1 μM), suggesting inaccuracies in the rate constants for reactions involving HOI, H_2_O_2_, and PAA (Figure S6). We performed additional experiments to redetermine the rate constant for the reaction between HOI and H_2_O_2_, resulting in an updated rate constant (*k*_HOI/OI-,H_2_O_2__) of 2.28 × 10^4^ M^–1^·s^–1^ at pH 7.1 (R9), about 3.6 times the previous value (6.23 × 10^3^ M^–1^·s^–1^)^13^ (Figure S7), as detailed in Text S4. Using this new rate constant and fitting the model to the oxidant changes under different iodide concentrations, the reaction rate constant between PAA and iodide, *k*_PAA,I^–^_, was determined to be (8.93 ± 1.75) × 10^2^ M^–1^·s^–1^ (R4), about 2.3 times of the previously reported value of 3.89 × 10^2^ M^–1^·s^–1^.^14^ We also found the reaction rate constant between PAA and HOI, *k*_PAA,HOI/OI-_, to be (7.38 ± 0.644) M^–1^·s^–1^ (R5), slower than the previously determined value of 15 M^–1^·s^–1^.^14^ Compared to the previous model, the updated kinetic model features a higher *k*_PAA,I^–^_, higher *k*_HOI/OI-,H_2_O_2__, and lower *k*_PAA,HOI/OI-_, resulting in increased HOI exposure during the first hour. This, in turn, may result in a higher formation of I-DBPs. With the newly determined *k*_HOI/OI-,H_2_O_2__, (R9), *k*_PAA,I^–^_, (R4), and *k*_PAA,HOI/OI-_ (R5) in Table 2, the experimental results fitted well to the kinetic model, as illustrated in Figure 4.

### Oxidant Kinetics and DBP Formation in the PAA/I^–^/NOM Systems

The oxidant changes in the PAA/I^–^/NOM systems were tested for SR-HA and MS-RO (Figure 5b,c). As Figure 5b,c shows, with a low NOM concentration (e.g., 5 mg/L), the decays of PAA and H_2_O_2_ were slightly inhibited, compared to those without NOM. When the NOM concentration was increased, the decays of PAA and H_2_O_2_ became ever slower, and a NOM concentration of 10 mg/L largely inhibited their decays. This is mainly because the formed HOI was primarily consumed by NOM, shutting down the recycling of iodide. Meanwhile, with an increasing NOM concentration, the formation of iodophenols at 2 s (the first sample taken for measurement immediately after mixing) was obviously decreased (Figure 5b,c), indicating the decreased concentration of [HOI + I^–^] at the very beginning to react with the phenol quencher. These results indicate that a fraction of NOM is very fast reactive to HOI, consuming the HOI immediately, leaving less [HOI + I^–^] recycled by PAA and H_2_O_2_, and causing the slowdown of the PAA and H_2_O_2_ decays.

**Figure 5:**
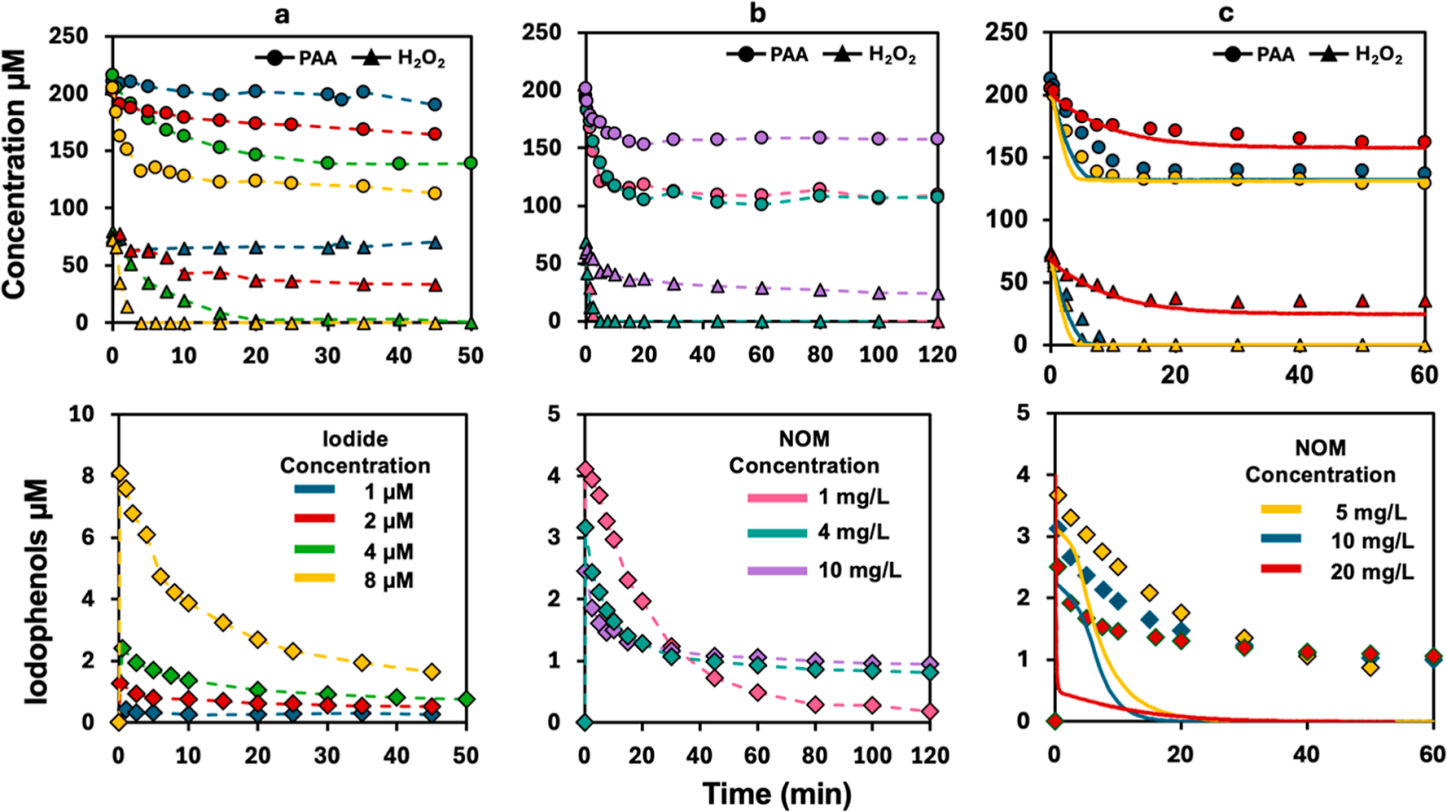
PAA/I^–^/NOM systems: temporal changes in PAA and H_2_O_2_ concentrations and the formation of iodophenols in sampling vials prespiked with 4 mM phenol in the presence of (a) varying concentrations of iodide and 10 mg/L SR-HA. (b) 4 μM iodide and varying concentrations of SR-HA, and (c) 4 μM iodide and varying concentrations of MS-RO. Experimental data are shown as symbols connected by dashed lines in panels (a,b), while in panel (c), experimental data are shown as symbols, and simulated results are shown in solid lines. All experiments used initial concentrations of 200 μM PAA and 70 μM H_2_O_2_ at pH 7.1 (10 mM phosphate buffer) and room temperature.

Furthermore, we noticed that, with the presence of NOM, even after an hour, there were still certain levels of iodophenols formed (Figure 5), which cannot be attributed to the reaction from HOI, because at the late stage, both PAA and H_2_O_2_ remained stable, indicating no free I^–^ or HOI present. Additional experiments were conducted to discern the underlying reasons. First, control experiments under the same reaction conditions (200 μM PAA, 70 μM H_2_O_2_, 4 μM I^–^, and 10 mg/L NOM), with samples taken over time into vials predosed with sodium thiosulfate, showed no formation of iodophenols. This confirmed that there were no significant background iodophenols formed in the PAA/I^–^/NOM system. Second, we mixed 4 μM freshly prepared HOI with NOM of different concentrations (5, 10, and 20 mg/L) and observed no formation of iodophenols in samples collected in vials prespiked with excess phenol after 5 min. This confirmed that HOI and NOM together will not generate detectable iodophenols or other long-lasting reactive iodine species. Third, to further confirm the accuracy of findings, another probe compound, 2,6-dibromophenol, was used to indicate HOI concentration by measuring the formation of 2,6-dibromo-4-iodophenol. With the different probe, similar results were found, with comparable levels of 2,6-dibromo-4-iodophenol formed (Figure S8). Based on the above results, we hypothesize that PAA, I^–^, and NOM together could generate some iodine reactive species, which can oxidize phenol to iodophenols, and 2,6-dibromophenol to 2,6-dibromo-4-iodophenols.

To evaluate our hypothesis, we tested the iodophenol formation during PAA disinfection with different concentrations of iodide (1–8 μM, Figure 5a) and NOM (1–10 mg/L, Figure 5b). With increased iodide concentration from 1, 2, 4, to 8 μM, the residual iodine reactive species, indicated by the iodophenol formation at the plateau phase, increased from 0.26, 0.52, 0.81, to 1.92 μM in the presence of 10 mg/L SR-HA NOM. An increase in the residual iodine reactive species was observed with increasing NOM dose from 1 to 10 mg/L at the same iodide dose of 4 μM. These results indicated that the formation of the reactive iodine species was dependent on both the iodide and NOM concentrations during PAA disinfection.

A recent study reported the formation of iodine radical species in the PAA/I^–^ system, though it was tested only under high iodide (up to 300 μM) and PAA (1 mM) concentrations.^38^ The reactive iodine radicals (e.g., I^•^, I_2_^•–^, and IO_3_^•^) were also found to generate in the peroxymonosulfate (PMS)/I^–^ system,^43^ while other studies have identified HOI as the dominant reactive species formed in the PMS/I^–^ systems.^44^ Because the unknown iodine species observed in the PAA/I^–^/NOM system decayed slowly and persisted for hours, they are unlikely to be free radicals. It is hypothesized that iodine radicals formed in the PAA/I^–^/NOM system could potentially react with NOM to form unknown reactive organo-iodine species, such as iodinated NOM intermediates or organo-I^+^ complexes that retain oxidizing power. However, further investigation is needed, particularly into the quantification of HOI and iodine radical species separately.

We made the effort to fit the experimentally measured oxidant changes in the presence of iodide and different NOM concentrations to the kinetic model for the MS-RO surrogate. As mentioned earlier, there is a small fraction of NOM fast reactive to HOI, consuming it almost immediately, as indicated by the rapid decrease in [HOI + I^–^] within 2 s in the presence of NOM. The concentration of this fast-reactive fraction (NOM_fast‑reactive_) was estimated by subtracting the iodophenol formation at 2 s from the initial iodide concentration (4 μM). The concentration of the bulk slow-reactive NOM was calculated by converting the total NOM concentration to a carbon basis (NOM_slow‑reactive_). With the assumed concentrations for the two fractions, we determined a *k*_(HOI, NOM fast‑reactive)_ of 6.35 × 10^6^ M^–1^·s^–1^ and a *k*_(HOI, NOM slow‑reactive)_ of 12.1 M^–1^·s^–1^ for the two fractions, by fitting the PAA and H_2_O_2_ decays under different NOM concentrations. Though both PAA and H_2_O_2_ decays fit relatively well to the model (Figure 5c), the experimental data and kinetic simulation of iodophenol formation did not align well because of the lack of reactions related to unknown reactive iodine species.

The HOI consumed by DBP precursors, also known as the total organic iodine (TOI), was estimated by subtracting the measured iodophenols formed (HOI + I^–^) and iodate (measured by IC) at any time point from the initial iodide concentration based on the mass balance (eq 6), shown in Figure 6a. In 4 h, the TOI was 2.69, 3.03, and 3.59 μM for MS-RO, SR-RO, and SR-HA NOM, respectively. For a total iodide concentration of 4 μM, the majority of HOI formed was consumed by NOM.

6 $$\left[TOI\right]=\left[I_{in}^{-}\right]-\left[I_{reactor}^{-}\right]-\left[{HOI}_{reactor}\right]-\left[{Iodate}_{reactor}\right]-\left[{ROI}_{reactor}\right],Unit:\mu M$$

where [I_in_^–^] is the initial iodide into the reactor, [HOI_reactor_] is the HOI concentration in the reactor, [Iodate_reactor_] is the iodate concentration in the reactor, and [ROI_reactor_] is the concentration of unknown iodine reactive species in the reactor. The sum of concentrations of I_reactor_^–^, HOI_reactor_, and ROI_reactor_ can be quantified by the formation of 2,6-dibromo-4-iodophenol using the 2,6-dibromophenol method at any given time.

**Figure 6:**
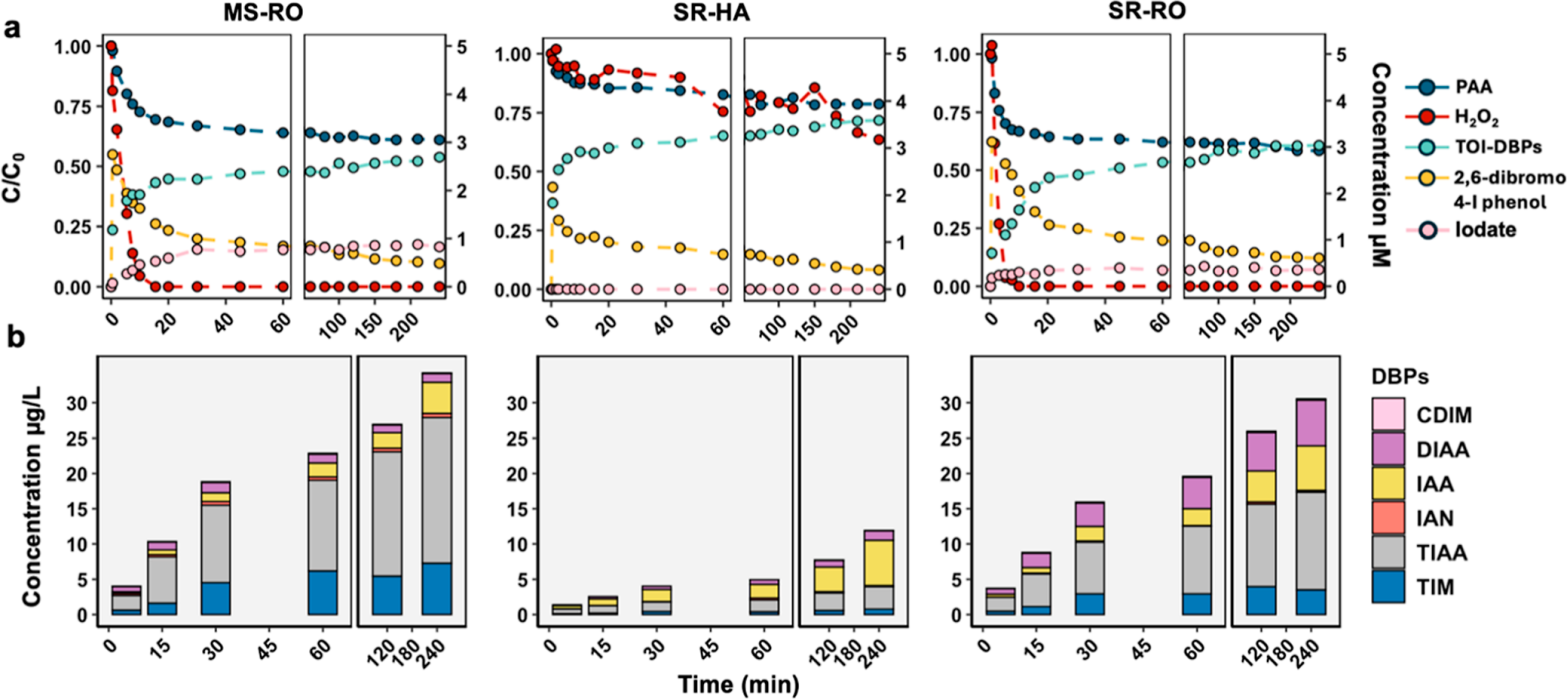
(a) The oxidant changes and (b) DBP formation in the PAA/I^–^/NOM systems. Different NOM surrogates, MR-RO, SR-HA, and SR-RO, at 20 mg/L were tested. Experimental data are shown as symbols connected by dashed lines in (a). All experiments used initial concentrations of 200 μM PAA, 70 μM H_2_O_2_, and 4 μM I^–^ at pH 7.1 (10 mM phosphate buffer) and room temperature.

The formation of DBPs in the PAA/I^–^/NOM systems was measured as shown in Figure 6b. A significant level of DBPs rapidly formed during the first 30 min, followed by a relatively slower formation of DBPs in the rest of 4 h. The total organic iodine from the known DBPs contributed to 8.7%, 6.6%, and 2.0% of the total HOI consumption for MS-RO, SR-RO, and SR-HA, respectively. MS-RO demonstrated the highest formation of known DBPs, although the HOI consumption was the lowest. In contrast, SR-HA featured the highest HOI consumption while yielding the lowest total known DBP formation. Thus, the majority of the TOI remained unknown. Recently, an increasing number of new iodinated DBPs have been identified, which could contribute to the unidentified TOI. Wang et al. used FT-ICR MS to identify the unknown iodinated DBPs and found that more than 68% of these I-DBPs were characterized as having aromatic or polycyclic aromatic structures.^45^ Similarly, Pan et al. identified several new polar aromatic iodinated DBPs, e.g., 3,5-diiodo-4-hydroxybenzaldehyde, 3,5-diiodosalicylaldehyde, 3,5-diiodosalicylic acid, and iodinated nitrophenols, which were detected in drinking water.^46^ Another recent study reported the identification of iodinated benzoquinones, including 2,6-diiodo-1,4-benzoquinone and 2-chloro-6-iodo-1,4-benzoquinone, in drinking water.^47^

As for the composition of the known DBPs, the tri-iodinated DBPs, TIAA and TIM, were the dominant species for MS-RO. As NOM becomes more hydrophobic, e.g., SR-RO and SR-HA, the formation of di- and tri-iodinated DBPs, e.g., TIM and DIAA, decreased, with monoiodoacetic acid becoming dominant. This trend is probably attributed to the higher reactivity of hydrophobic NOM with HOI, as the precursors for iodinated DBPs are largely aromatic and polycyclic.^45^ The higher the hydrophobic fraction, the more intense the competition between NOM for the limited HOI, thereby suppressing the formation of di- or trihalogenated DBPs. The DBP composition was relatively consistent in samples collected at different times, indicating the limited transformation of DBP species in the PAA/I^–^/NOM system once they were formed. Based on the HOI exposure and DBP formation results, it is highly recommended to keep the PAA disinfection time within 15 min in iodide-containing waters, which can effectively limit the iodinated DBP formation.

### Effect of H_2_O_2_ Concentration on Oxidant Kinetics and DBP Formation

Kinetically, H_2_O_2_ can significantly impact HOX exposure by rapidly reducing HOX back to halides. To test whether DBP formation can be controlled by increasing the H_2_O_2_ concentration, MS-RO NOM at 20 mg/L was used to examine the effect of an elevated H_2_O_2_ concentration (i.e., PAA/H_2_O_2_ ratio of 1:2 (200:400 μM)), with the results shown in Figure 7.

**Figure 7:**
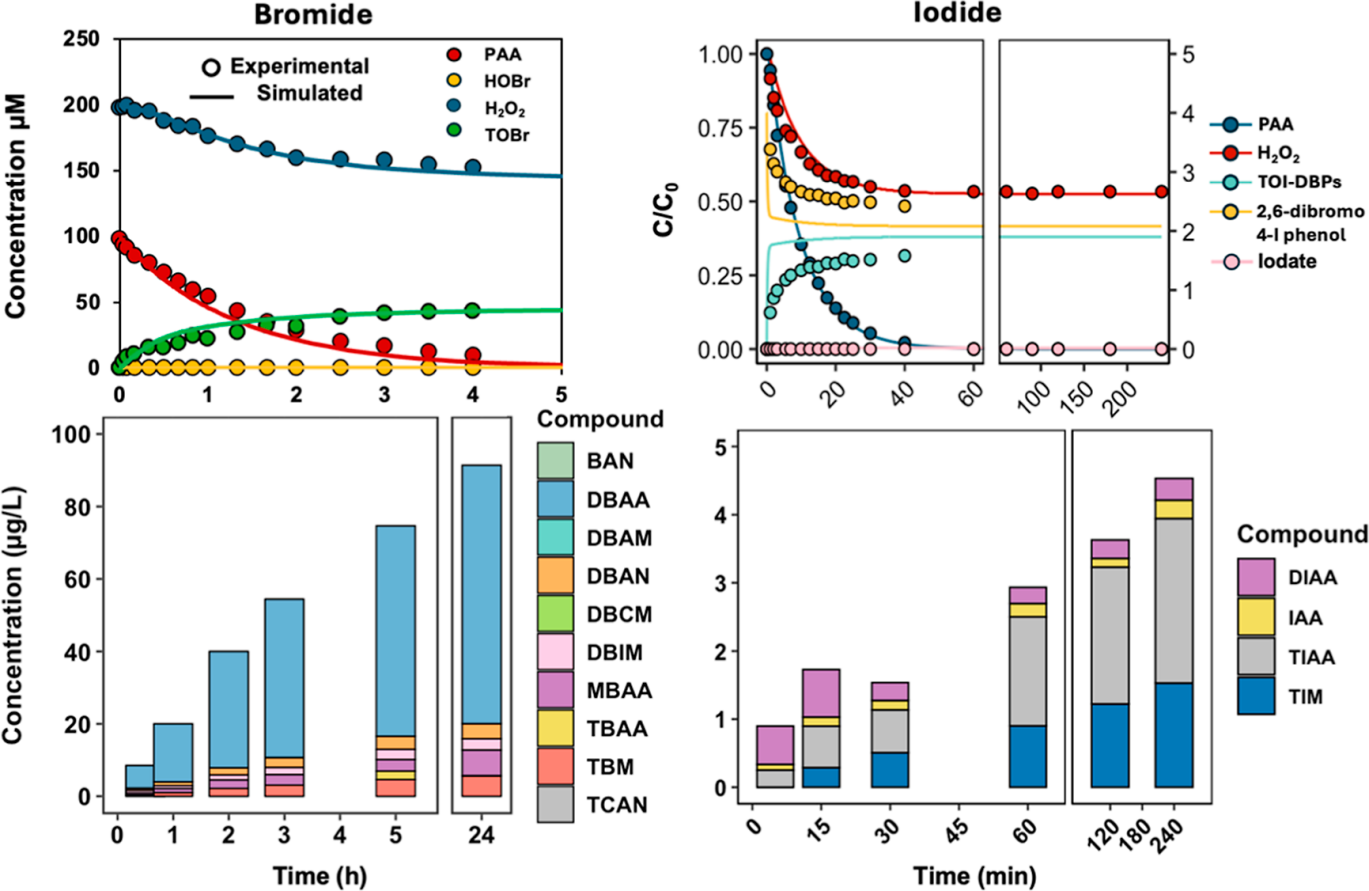
Oxidant changes (top) and DBP formation (bottom) during PAA disinfection at a PAA/H_2_O_2_ molar ratio of 1:2 in the presence of bromide (left) or iodide (right) and MS-RO NOM at 20 mg/L. For the oxidant decay plots, experimental measurements are shown as symbols, and kinetic model simulations are shown as solid lines. All experiments used the initial concentrations of 200 μM PAA, 400 μM H_2_O_2_, and 1 mM Br or 4 μM I^–^, at pH 7.1 (10 mM phosphate buffer) and room temperature.

For the bromide system, PAA decay was similar to that under a low H_2_O_2_ dose (60 μM, Figure 3) since it was not affected significantly by H_2_O_2_ concentration due to the excessive bromide concentration (1.0 mM). In contrast, the H_2_O_2_ decay was slowed down compared to that without NOM based on simulation (Figure S9), and no detectable HOBr was observed over the 4 h reaction time. Notably, the oxidant changes predicted using our refined kinetic model aligned well with the experimental data. The TOBr formed at 5 h was 43.3 μM, calculated based on eq 5 and 41.7 μM based on model prediction, which is approximately one-third lower than that (61.7 μM) when the H_2_O_2_ concentration was 60 μM. As for DBPs, similar to the low H_2_O_2_ case, the DBP formed gradually within 5 h, with no significant increase in 24 h. The total DBP formation was 91.98 μg/L, much lower than the 586.5 μg/L when H_2_O_2_ was 60 μM. The known DBPs contributed to only 2.1% of predicted TOBr formation, indicating that more products remained unknown.

For the iodide system (4 μM iodide), PAA exhibited continuous decay to complete depletion within 40 min. H_2_O_2_ decreased gradually to approximately 50% of its initial concentration over the same period and subsequently remained stable. No iodate formation was detected under this condition. The TOI concentration was determined to be 1.51 μM in 30 min using eq 6, which is approximately half of the TOI formed when the H_2_O_2_ concentration was 70 μM. Furthermore, the higher H_2_O_2_ concentration substantially suppressed the formation of known DBPs. The TIM and TIAA concentrations decreased from 7.27 and 20.64 μg/L to 1.52 and 2.41 μg/L, respectively, when the H_2_O_2_ concentration was increased from 70 μM to 400 μM. Results from other studies also reported a lower formation of I-DBPs when water samples were treated by PAA solutions with a higher H_2_O_2_-to-PAA molar ratio (PAA/H_2_O_2_ < 1).^13,48^ Overall, a higher H_2_O_2_ concentration can inhibit the brominated- and iodinated-DBP formation by limiting the HOX availability to the slow- or relatively fast-reactive fractions of NOM. However, it will have a less pronounced effect on DBP formation from the fast-reactive fraction of NOM.

## Environmental Significance

Overall, in this study, we monitored the kinetics of oxidant changes specifically and measured DBP formation in the PAA/halide/NOM systems. We have further refined and improved the kinetic model that can accurately simulate both the PAA/Br^–^ and PAA/Br^–^/NOM systems in good agreement with experimental data. The NOM that is fast-reactive with HOBr primarily contributes to the DBP formation, with the rapid formation of brominated DBPs from all the tested NOM surrogates. HAAs, particularly dibromoacetic acid (DBAA), constitute the majority of the detected DBPs, which differed significantly from those formed during direct bromination. The identified DBPs, however, accounted for a small fraction of the consumed total bromide, suggesting that most brominated DBPs remain unidentified. At a PAA/H_2_O_2_ molar ratio of 1:2, the formation of known DBPs was decreased by approximately 90%, while TOBr concentration was reduced by one-third.

For I^–^-containing waters treated with PAA (i.e., the PAA/I^–^/NOM systems), the kinetic model fits the experimental measurements well after experimentally redetermining the rate constants of key reactions with iodide (*k*_HOI/OI-,H_2_O_2__, *k*_PAA,I^–^_, and *k*_PAA,HOI/OI-_). Unidentified reactive iodine species were formed and persisted in the system, with their concentrations dependent on both iodide and NOM levels. A small fraction of NOM can react immediately with HOI, leading to the rapid consumption of iodide initially. The majority of NOM reacts with HOI more slowly, which can be inhibited by an increasing H_2_O_2_ concentration. A PAA/H_2_O_2_ molar ratio of 1:2 effectively reduced 44.23% of TOI and 85.16% of iodinated DBPs, compared to a PAA/H_2_O_2_ molar ratio of 1:0.35, with TIM and TIAA identified as the dominant DBPs.

For practical applications of PAA disinfection in the presence of halides, DBP formation is strongly influenced by the presence of NOM fractions that are highly reactive with HOBr and HOI. In source waters rich in hydrophobic NOM, coagulation or other pretreatment strategies are recommended to be implemented to reduce these fractions prior to PAA application. It is recommended to keep the PAA disinfection time within 15 min to largely reduce brominated and iodinated DBPs. However, limiting the contact time to reduce DBP formation may conflict with the CT (concentration × time) values required for effective pathogen log-inactivation, especially for more resistant microorganisms. For example, a PAA dosage of 5.5–6 mg/L (59–79 μM) and a contact time of 1 h is needed for 99.99% inactivation of *Escherichia coli*.^49^ Similarly, 5–10 mg/L PAA (65–131 μM) for 15 min is required to inactivate more than 95% of total and fecal coliforms when treating secondary effluent.^50^ Thus, employing PAA formulations with a lower PAA/H_2_O_2_ ratio is recommended as a more applicable solution to suppress the DBP formation. Finally, this work has been conducted mainly at pH 7.1, and the kinetic model treats HOX/OX^–^ speciation collectively at the set pH. However, considering that the p*K*_a_ values of PAA, HOBr, and HOI at 8.2, 8.7, and 10.4 are relatively weak, the major findings as well as the refined kinetic model of this work are expected to remain valid within a moderate change of water pH conditions.

## Supplementary Material



## Notes

Additional text, including chemicals and reagents, mechanism for DPD-KI and HRP-ABTS methods, detailed methods for DBP extraction, experiments on determination of rate constant between HOI and H_2_O_2_; tables, including NOM surrogate properties; and figures, including validation of oxidant testing methods, HOBr decay during bromination, oxidants [PAA, H_2_O_2_, and HOX] changes with/without NOM in PAA/halide system, and rate determination for the reaction between HOI and H_2_O_2_ (PDF)
