# Supplementary material for: Kinetics of Hypohalous Acid Intermediates Governing Disinfection Byproduct Formation in Peracetic Acid-Treated Halide-Containing Waters
Source: Environ Sci Technol. 2026 Jan 8;60(2):2173–85. doi: 10.1021/acs.est.5c12123 (PMC12825158; doi:10.1021/acs.est.5c12123)
Supplement: Supplementary file 1 [file es5c12123_si_001.pdf]

Supporting Information for

Kinetics of Hypohalous Acid Intermediates Governing Disinfection Byproduct Formation in Peracetic Acid–Treated Halide-Containing Waters

Jiaqi Li,[†] Samantha DiLoreto,[†] and Ching-Hua Huang[†,*]

[†]School of Civil and Environmental Engineering, Georgia Institute of Technology, Atlanta, Georgia, United States

*Corresponding author Email: ching-hua.huang@ce.gatech.edu

19 pages, including 4 texts, 4 tables, 9 figures, and supporting references

Table of Contents

Text S1. Chemicals and reagents 4

Table S1. Elemental composition for the selected NOM surrogates. 5

Text S2. Validation of methods used in this study for oxidant measurement 10

Figure S1. Calibration curves for PAA, HOI, and HOBr using the KI-DPD method. A(515nm) = absorbance at 515 nm. 6

Figure S2. The linear relationship between the theoretical concentration and measured concentration for PAA and H_2O_2 using the HRP-ABTS method, and for HOI using ABTS with/without the addition of the HRP catalyst. 7

Figure S3. The calibration curve for HOBr using the ABTS-H_2SO_4 method: the absorbance at 415 nm (A(415nm)) for HOBr solutions of varying concentrations with or without the presence of 50 μM PAA. Note: 18.5 μM H_2O_2 coexisted with 50 μM PAA in the tested solutions. 8

Figure S4. The linear relationship between the theoretical concentration and measured concentration for HOI using the phenol method. 9

Text S3. Extraction and analysis of DBPs 10

Table S2. The calculated overall SD for each experimental test...12

Table S3. Summary of apparent reaction rate constant for model compounds exhibiting high reactivity with HOBr 13

Table S4. Summary of apparent reaction rate constant for model compounds exhibiting moderate reactivity with HOBr...13

Figure S5. The decay of HOBr during direct bromination for MS-RO NOM. Experimental conditions: 90 μM HOBr, 20 mg/L MS-RO, and no NOM for the control, 10 mM phosphate buffer at pH 7.1, room temperature, in amber glass reactor...14

Figure S6. Temporal changes in the concentrations of PAA (blue), H_2O_2 (red), IO_3^- (green), and HOI (yellow) in the PAA/I^- systems based on experimentally measured values (dots) and kinetic model simulations using a previously developed model (solid lines).[6] All experiments used the

same initial concentrations of PAA (200 μM) and H_2O_2 (74 μM), with varying iodide concentrations as indicated in the plot, and were conducted at pH 7.1 (10 mM phosphate buffer) and room temperature..15

Text S4. Determination of the reaction rate constant between PAA and HOI............................16

Figure S7. The fitting of experimental data to the integrated reaction rate equation (1Ct-1C0=kHOI,H2O2t) for reaction HOI + H_2O_2 → I^-. The H_2O_2 and HOI concentrations are both 1μM for the left plot and 2 μM for the right plot. Experimental conditions: 10 mM phosphate buffer at pH = 7.1, room temperature, in dark...16

Figure S8. Changes in PAA and H_2O_2 concentrations (Left) and the formation of iodophenols or dibromoiodophenols in vials pre-spiked with 4 mM phenol/dibromorphenol (Right) over time in the presence of 4 μM iodide and of (a) 5 mg/L Suwannee River humic acid NOM, and (b) 20 mg/L Mississippi River-RO NOM. Reaction conditions: 200 μM PAA, 70 μM H_2O_2, 4 μM iodide, 10 mM phosphate buffer (pH 7.1), at room temperature...17

Figure S9. Simulated PAA (blue), H_2O_2 (yellow) and HOBr (yellow) concentrations change over time with 100 μM PAA, 200 μM H_2O_2, and 1 mM bromide. 18

References 19

Text S1. Chemicals and Reagents

Sodium hydroxide (NaOH), sulfuric acid (H_2SO_4), sodium thiosulfate ($Na_2S_2O_3$), sodium hydrogen phosphate (Na_2HPO_4), *N*,*N*-diethyl-p-phenylenediamine (DPD), sodium hypochlorite (NaOCl), phenol, 2-iodophenol, 4-iodophenol, sodium chloride (NaCl), sodium bromide (NaBr), potassium iodide (KI), potassium bromate ($KBrO_3$), potassium iodate (KIO_3), ammonium nitrate (NH_4NO_3), formic acid, acetic acid, sodium sulfate, 1,2-dibromopropane, methyl *tert*-butyl ether (MtBE) were purchased from Sigma-Aldrich or Fisher Scientific (Fair Lawn, NJ). Deionized water (DI water) (>18 mΩ-cm) was produced from a Milli-Q water purification system (Billerica, MA). Natural organic matter (NOM) surrogates were purchased from the International Humic Substance Society (IHSS) with detailed elemental composition shown below obtained online.

Chloroform, bromodichloromethane, bromoform, dibromochloromethane, chloropropanone, 1,1,1-trichloropropanone, chloroacetonitrile, trichloroacetonitrile, dichloroacetonitrile, bromoacetonitrile, iodoacetonitrile, dichloroacetamide, monochloroacetic acid , monobromoacetic acid, dichloroacetic acid, dibromoacetic acid, trichloroacetic acid, bromochloroacetic acid, bromodichloroacetic acid, chlorodibromoacetic acid,tribromoacetic acid,iodoacetic acid, decafluorobiphenyl , 2-bromobutanoic acid, 1,2-dibromopropane were purchased from Sigma-Aldrich.

Dichloroiodomethane, bromochloroiodomethane, dibromoiodomethane, chlorodiiodomethane, bromodiiodomethane, iodoform, 1,1-dichloropropanone, Bromochloroacetonitrile, dibromoacetonitrile, bromochloroacetamide, trichloroacetamide, dibromoacetamide, diiodoacetic acid, triiodoacetic acid were obtained from Toronto Research Chemicals.

Table S1. Elemental composition for the selected NOM surrogates.

NOM		Suwannee River Humic Acid	Suwannee River RO	Mississippi River RO
Cat. No.		2S101H	1R101N	1R110N
Elemental Composition	H_2O	20.4	8.15	8.55
	Ash	1.04	7	8.05
	C	52.63	52.47	49.98
	H	4.28	4.19	4.61
	O	42.04	42.69	41.4
	N	1.17	1.1	2.36
	S	0.54	0.65	2.62
	P	0.013	0.02	nd
Acid functional groups	*Carboxyl*	9.13	9.85	12.43
	Phenolic	3.72	3.94	0.83
DOC concentration mgC/mg		0.46	0.43	0.42
DON concentration mgN/mg		0.0774	0.0794	0.0898

Text S2. Validation of methods used in this study for oxidant measurement.

The KI-DPD method. To 3 mL sample with PAA concentrations ranging from 2 to 20 μM after appropriate dilution if needed, 30 μL KI (10 g/L), 150 μL DPD indicator solution (1 g/L), and 150 μL phosphate buffer (0.5 M, pH 7.0) were added in sequence. The sample was treated with an excess of potassium iodide, resulting in the liberation of iodine, which subsequently reacted with DPD to yield a pink-colored chromophore.

For PAA concentration ranging from 2.5 -20 μM, the absorbance of this species at 515 nm was directly proportional to the PAA concentration. Solutions of HOI and HOBr ranging from 2.5 to 20 μM were prepared and immediately analyzed. The slopes (~0.012) of calibration curves obtained for HOBr and HOI were similar to that of PAA, indicating an identical stoichiometric reaction with KI-DPD (**Figure S1**). Consequently, the absorbance at 515 nm represents the total concentration of PAA and HOX in PAA-halide systems.

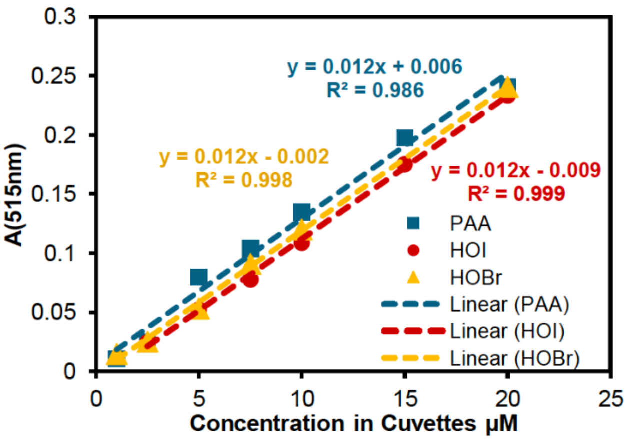

Figure S1. Calibration curves for PAA, HOI, and HOBr using the KI-DPD method. A(515nm) = absorbance at 515 nm.

The HRP- ABTS method. For samples with total oxidant concentrations below 30 μM, 2.7 mL was transferred into a cuvette, followed by the addition of 30 μL of 200 mg/L HRP and 240 μL of 1 mM ABTS solution. The absorbance was then recorded at 415 nm. Mechanistically, H_2O_2 oxidizes the ferric heme peroxidase (Por–Fe^{3+}) to Compound I, a porphyrin π-cation

radical containing Fe(IV).[1] Compound I is subsequently reduced via two one-electron steps through Compound II, while ABTS is oxidized to $ABTS^{\bullet +}$. Overall, one mole of H_2O_2 yields two moles of $ABTS^{\bullet +}$. PAA behaves similarly to H_2O_2 in the HRP-ABTS method.

With the HRP-ABTS method, PAA, H_2O_2, HOBr, and HOI were each observed to oxidize ABTS to its radical form ($ABTS^{\bullet +}$) in the presence of HRP at a 1:2 molar ratio, i.e., one mole of oxidant can oxidize two moles of ABTS. Notably, HOI could directly oxidize ABTS to $ABTS^{\bullet +}$ even without HRP (**Figure S2**). Thus, concentrations measured by the HRP-ABTS method represent the sum of [PAA + H_2O_2 + HOX]. Hence, the H_2O_2 concentration was determined by subtracting the combined [PAA + HOX] concentration measured by the KI-DPD method from the total oxidant concentration obtained via the HRP-ABTS method.

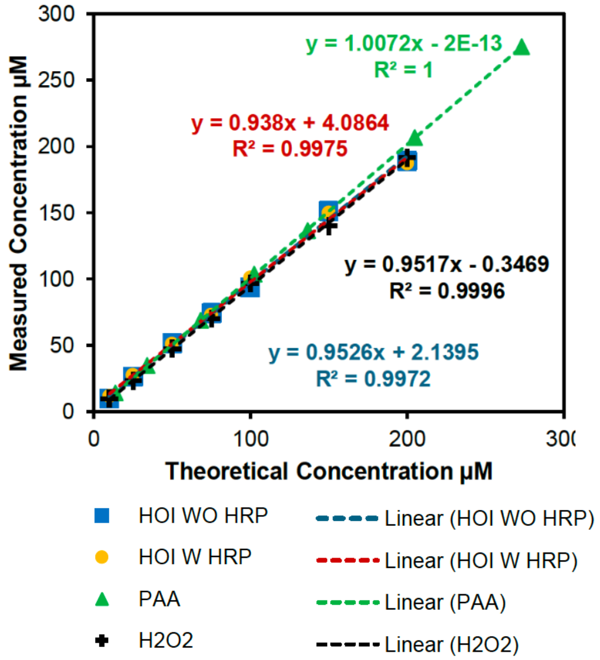

Figure S2. The linear relationship between the theoretical concentration and measured concentration for PAA and H_2O_2 using the HRP-ABTS method, and for HOI using ABTS with/without the addition of the HRP catalyst.

The H_2SO_4-ABTS method. The HOBr concentration was specifically measured using the H_2SO_4-ABTS method. To 3 mL sample, 120 μL of 50 mM sulfuric acid and 80 μL of 4 mM ABTS were added, followed by an immediate measurement of absorbance at 415 nm. For HOBr solution ranging from 2.5 to 20 μM, it was found that PAA (50 μM) and H_2O_2 (18.5 μM) did not interfere with accurate HOBr quantification, confirming the suitability of the H_2SO_4-ABTS method for quantifying HOBr in the oxidant mixtures (**Figure S3**). This is because ABTS can rapidly react with HOBr in acidic conditions but reacts rather slowly with PAA and H_2O_2 directly. Also, the reaction between Br^- and PAA is relatively slow and thus does not interfere with the immediate measurement of HOBr concentration. Therefore, the PAA concentration can be determined indirectly by subtracting the HOBr concentration from the total concentration of PAA and HOX measure by the KI-DPD method.

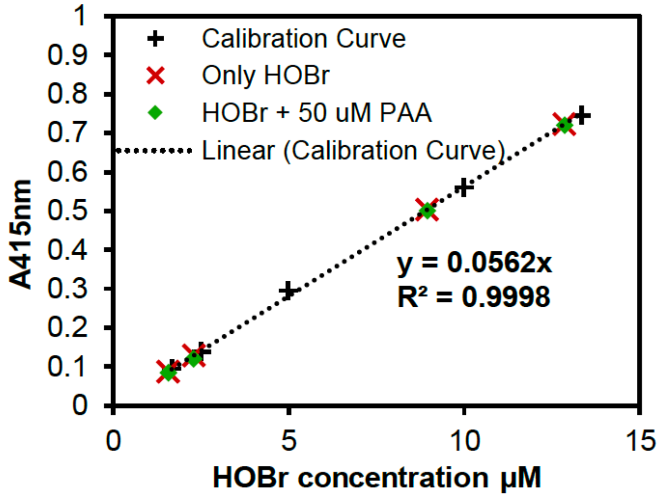

Figure S3. The calibration curve for HOBr using the ABTS-H_2SO_4 method: the absorbance at 415 nm (A(415nm)) for HOBr solutions of varying concentrations with or without the presence of 50 μM PAA. Note: 18.5 μM H_2O_2 coexisted with 50 μM PAA in the tested solutions.

The Iodophenols Formation Method. As for PAA/iodide systems, the sums of concentrations of [PAA + HOI] and [PAA+ H_2O_2 +HOI] were determined by the same methods mentioned above, KI-DPD and HRP-ABTS, respectively. To measure HOI specifically, samples were concurrently collected into HPLC vials pre-spiked with 4 mM phenol or 2,6-dibromophenol, which rapidly quenched HOI but minimally reacted with PAA. Residual iodide is oxidized to HOI continuously by PAA in the solution; however, the HOI formed can be quantified by the phenol method via immediate quenching of HOI and formation of iodophenols (**Figure S4**). The formation of 2- and 4-iodophenols (from phenol) or 2,6-dibromo-4-iodophenol (from 2,6-dibromophenol) was measured using HPLC with a SC-C18 column. The HPLC mobile phase consisted of 65% methanol and 35% water at a flow rate of 0.5 mL/min. Additionally, it has been reported that iodate and iodide ions could form triiodide at higher acidity which can then react with phenol and form iodophenol.[34] We performed control experiments and confirmed negligible formation of iodophenols when 4 μM iodate, 4 μM iodide, and 4 mM phenol were mixed at pH 7.1, indicating slow iodate-iodide reactions under most experimental conditions of this study. Hence, the PAA concentration was determined indirectly by subtracting the HOI concentration from the total concentration of PAA and HOI.

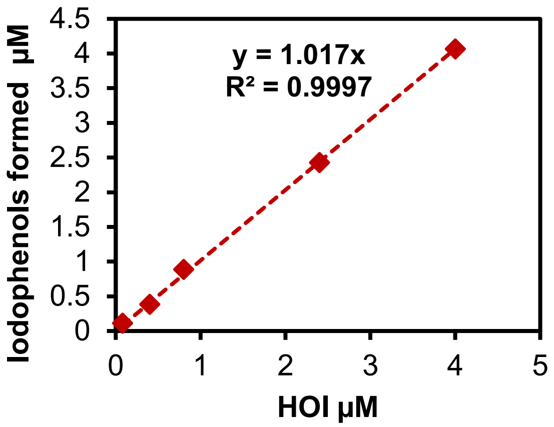

Figure S4. The linear relationship between the theoretical concentration and measured concentration for HOI using the phenol method.

Text S3. Extraction and Analysis of DBPs

EPA 551 Method. DBPs were extracted using liquid-liquid extraction (LLE) with methyl *tert*-butyl ether (MTBE). Briefly, 50 mL of sample was transferred to a 60-mL amber glass vial, and decafluorobiphenyl (10 ppb) was added as a surrogate standard. MTBE (3 mL) was then added, followed by 10 g of sodium sulfate. The mixture was shaken vigorously for 4 minutes, and the MTBE layer was transferred to a GC vial for analysis. Samples were analyzed using gas chromatography with electron capture detection (GC-ECD; 7890A GC System, Agilent Technologies, USA) equipped with a J&W DB-1 column (30 m × 0.25 mm × 0.25 μm). The oven temperature program was as follows: 35 °C for 15 min, ramped to 100 °C at 10 °C/min and held for 2 min, then increased to 150 °C at 5 °C/min and held for 3 min. Injector and detector temperatures were set to 230 °C and 260 °C, respectively. High-purity nitrogen served as both carrier and make-up gas. QA/QC procedures followed those described by the EPA 551 Method

EPA 552 Method. HAAs were extracted by LLE followed by acid derivatization. A 40-mL sample was placed in a 60-mL amber vial, and 2-bromobutanoic acid (10 ppb) was added as a surrogate. Then, 18 g sodium sulfate was added, and the sample was shaken until sodium sulfate was mostly dissolved. Afterwards, 4 mL of MTBE containing 1,2-dibromopropane (100 ppb, internal standard) was added and shaken vigorously for 3 minutes. The MTBE layer (~3 mL) was transferred to a 15 mL centrifuge tube. Next, 3 mL of 10% sulfuric acid in methanol was added, and samples were heated at 50 ± 2 °C for 2 hours. After cooling to room temperature, 7 mL of a 150 g/L sodium sulfate solution was added and vortexed. The aqueous layer was discarded. Then, 1 mL of saturated sodium bicarbonate was added, followed by vortexing multiple times, and the organic layer was transferred to a GC vial. Samples were analyzed using the same GC-ECD system and column as described above. The oven program was: 40 °C for 5 min, ramped to 52 °C at 5 °C/min and held for 5 min, then to 80 °C at 2.5 °C/min (held for 2 min), next to 150 °C at 10 °C/min (held for 2 min), and finally to 185 °C at 25 °C/min.

Injector and ECD temperatures were 210 °C and 280 °C, respectively. Nitrogen was used as the carrier and make-up gas. QA/QC procedures followed those described by the EPA 552 Method.

Table S2. The calculated overall SD for each experimental test.

System	PAA μM	H_2O_2 μM	Iodide μM	Bromide mM	NOM	Overall SD
PAA/Br^-	100	40	0	1	0	2.01
	100	60	0	1	0	13.34
	100	40	0	2	0	5.49
PAA/Br^- /NOM	100	40	0	1	10 ppm SR-RO	7.45
	100	40	0	1	20 ppm SR-RO	6.02
	100	40	0	1	10 ppm MS-RO	6.50
	100	40	0	1	20 ppm MS-RO	3.36
	100	40	0	1	10 ppm SR-HA	9.23
	100	40	0	1	20 ppm SR-HA	5.09
	100	200	0	1	20 ppm MS-RO	3.75
PAA/I^-	200	74	0.3	0	0	6.88
	200	74	0.7	0	0	6.48
	200	74	1	0	0	6.79
	200	74	2	0	0	6.11
	200	74	4	0	0	2.94
	200	74	10	0	0	8.61

The kinetic modeling for oxidant changes in the $PAA/I^-/NOM$ system was not successful. Thus, no overall standard deviation between experimental and simulated results was calculated for experiments in the $PAA/I^-/NOM$ system.

Table S3. Summary of apparent reaction rate constant for model compounds exhibiting high reactivity with HOBr

Compound	$k_{(HOBr/OBr-)}$ $M^{-1}s^{-1}$	Ref
4-Nitrophenol	3.6×10^6	[3, 4]
Resorcinol	9.0×10^6	[3]
Phloroglucinol	1.4×10^7	[3]
Hesperetin	8.2×10^6	[3]
3-Methoxyphenol	2.1×10^6	[3, 4]
3-Chlorophenol	1×10^6	[3, 4]
4-Nitro-*m*-cresol	7.5×10^5	[3]

Table S4. Summary of apparent reaction rate constant for model compounds exhibiting moderate reactivity with HOBr

Compound	$k_{(HOBr/OBr-)}$ $M_{-1}s_{-1}$	Ref
Phenol	0.8×10^4	[3]
p-Aminophenol	8.9×10^4	[3]
4-Methoxyphenol	3.1×10^4	[3]
4-Chlorophenol	3.2×10^4	[3]
Tannic Acid	1.7×10^5	[5]
Gallic acid	1.5×10^4	[5]
Hydroquinone	6×10^4	[5]
4-Acetylphenol	1.1×10^5	[3, 4]
o-Cresol (2-methylphenol)	4.5×10^4	[3, 4]
Average	4.8×10^4	

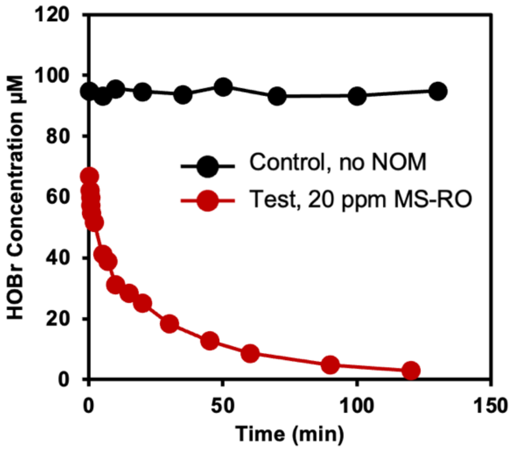

Figure S5. The decay of HOBr during direct bromination for MS-RO NOM. Experimental conditions: 90 µM HOBr, 20 mg/L MS-RO, and no NOM for the control, 10 mM phosphate buffer at pH 7.1, room temperature, in amber glass reactor.

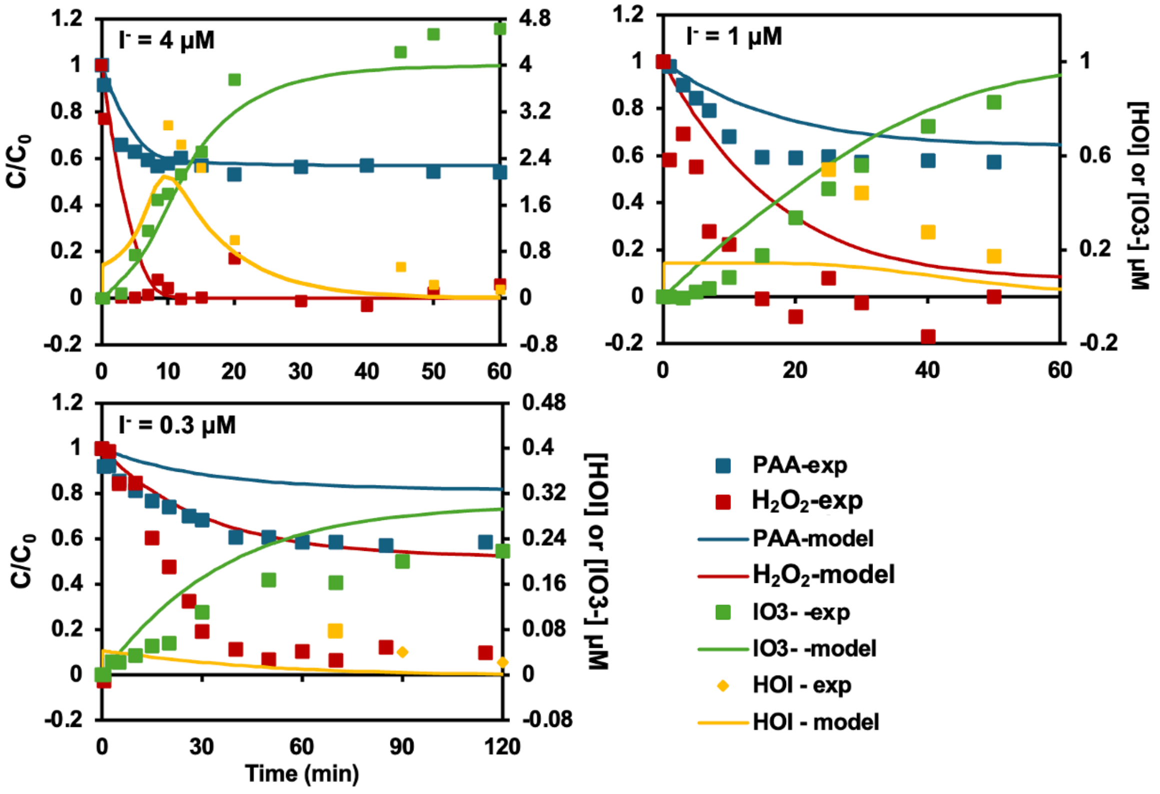

Figure S6. Temporal changes in the concentrations of PAA (blue), H_2O_2 (red), IO_3^- (green), and HOI (yellow) in the PAA/I^- systems based on experimentally measured values (symbols) and kinetic model simulations using a previously developed model (solid lines).[6] All experiments used the same initial concentrations of PAA (200 μM) and H_2O_2 (74 μM), with varying iodide concentrations as indicated in the plot, and were conducted at pH 7.1 (10 mM phosphate buffer) and room temperature.

Text S5. Determination of the reaction rate constant between PAA and HOI

Experiments were conducted by mixing HOI and H_2O_2 at equal concentrations (both 1 μM or both 2 μM) in a reactor containing 10 mM phosphate buffer (pH 7.1). For each experiment, a 100 μM HOI stock solution was freshly prepared and immediately quantified using the KI-DPD method. During the reaction, aliquots of the mixture were collected at specific times into HPLC vials pre-spiked with 4 mM phenol. The iodophenols formed were then analyzed using HPLC. The reaction rate of HOI with H_2O_2 is described by Equation S2, which can be integrated into a linearized form (Equation S3). By fitting the experimental data to the integrated rate equation (Figure S7), the average second-order rate constant $k_{HOI,H2O2}$ was calculated to be 2.28×10^4 $M^{-1}s^{-1}$, which is higher than the previously reported value (6.23×10^3 $M^{-1}s^{-1}$).

$$\frac{d[HOI]}{dt} = -k_{HOI,H2O2}[HOI][H_2O_2] \approx -k_{HOI,H2O2}[HOI][HOI] = -k_{HOI,H2O2}[HOI]^2 \quad (S2)$$

$$\frac{1}{C_t} - \frac{1}{C_0} = k_{HOI,H2O2}t \quad (S3)$$

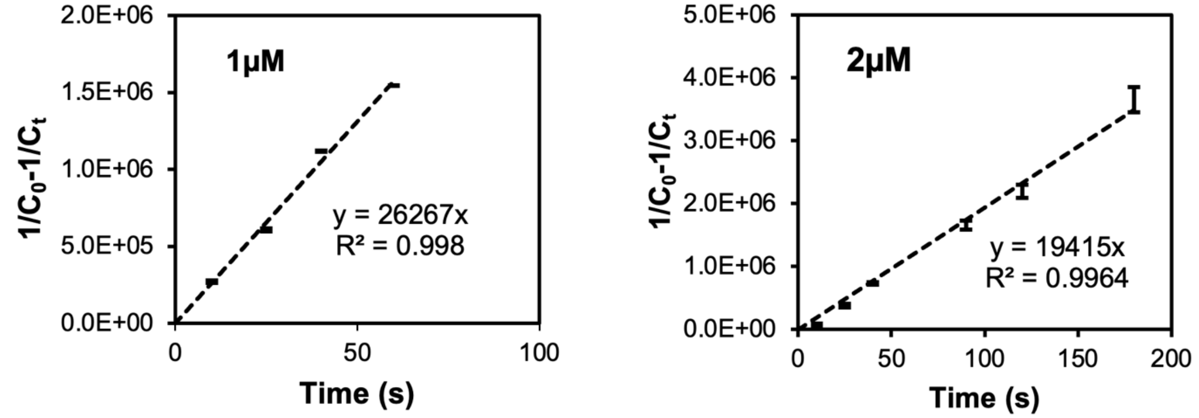

Figure S7. The fitting of experimental data to the integrated reaction rate equation ($\frac{1}{C_t}$-$\frac{1}{C_0}$=$k_{HOI,H2O2}t$) for reaction HOI + $H_2O_2 \rightarrow I^-$. The H_2O_2 and HOI concentrations are both 1μM for the left plot and 2 μM for the right plot. Experimental conditions: 10 mM phosphate buffer at pH = 7.1, room temperature, in dark.

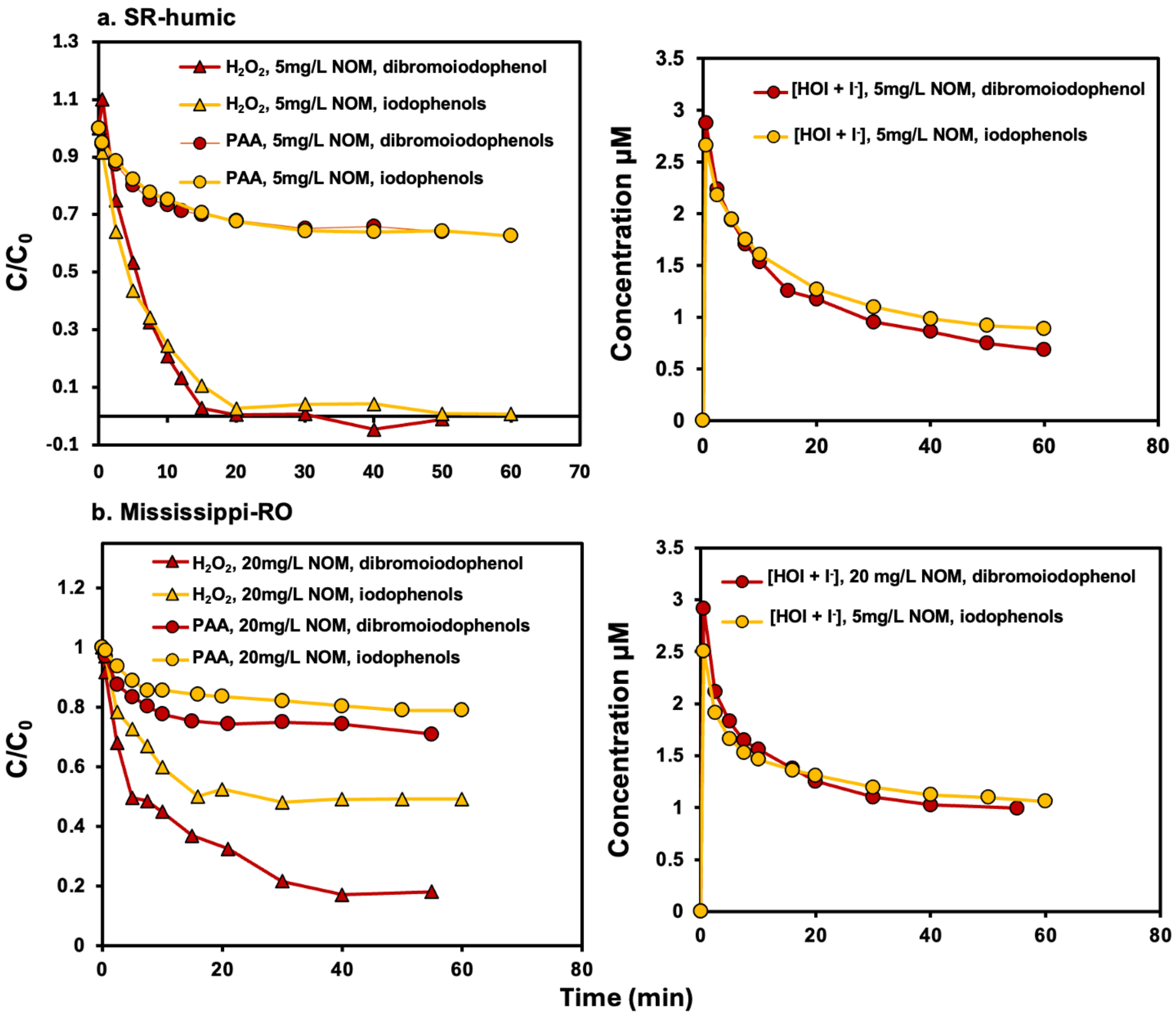

Figure S8. Changes in PAA and H_2O_2 concentrations (Left) and the formation of iodophenols or dibromoiodophenols in vials pre-spiked with 4 mM phenol/dibromorphenol (Right) over time in the presence of 4 μM iodide and of (a) 5 mg/L Suwannee River humic acid NOM, and (b) 20 mg/L Mississippi River-RO NOM. Reaction conditions: 200 μM PAA, 70 μM H_2O_2, 4 μM iodide, 10 mM phosphate buffer (pH 7.1), at room temperature.

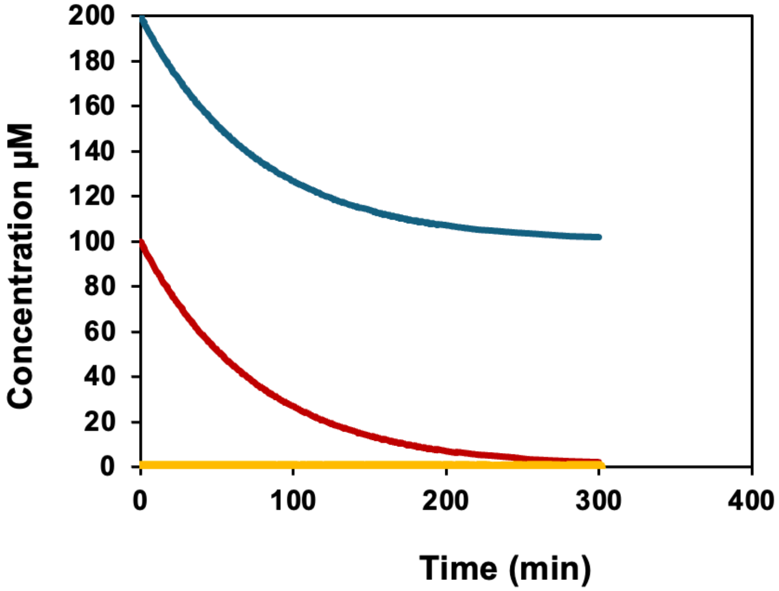

Figure S9. Simulated PAA (blue), H_2O_2 (yellow) and HOBr (yellow) concentrations change over time with 100 μM PAA, 200 μM H_2O_2, and 1 mM bromide.

References

1. Wang, J. Y.; Kim, J.; Li, J. Q.; Krall, C.; Sharma, V. K.; Ashley, D. C.; Huang, C.-H., Rapid and Highly Selective Fe(IV) Generation by Fe(II)-Peroxyacid Advanced Oxidation Processes: Mechanistic Investigation via Kinetics and Density Functional Theory. *Environ. Sci. Technol.* **2024,** *58* (38), 17157-17167.
2. Zhang, T.; Huang, C.-H., Modeling the Kinetics of UV/Peracetic Acid Advanced Oxidation Process. *Environ. Sci. Technol.* **2020,** *54* (12), 7579-7590.
3. Guo, G.; Lin, F., The bromination kinetics of phenolic compounds in aqueous solution. *J. Hazard. Mater.* **2009,** *170* (2), 645-651.
4. Heeb, M. B.; Criquet, J.; Zimmermann-Steffens, S. G.; von Gunten, U., Oxidative treatment of bromide-containing waters: Formation of bromine and its reactions with inorganic and organic compounds — A critical review. *Water research (Oxford)* **2014,** *48*, 15-42.
5. Echigo, S.; Minear, R. A.; Matsui, S.; Matsuda, T.; Shimizu, Y.; Tanaka, H., Kinetics of the reaction of hypobromous acid and organic matters in water treatment processes. *Water Science and Technology* **2006,** *53* (11), 235-243.
6. Wang, J. Y.; Xu, J. L.; Kim, J.; Huang, C.-H., Mechanistic Insight for Disinfection Byproduct Formation Potential of Peracetic Acid and Performic Acid in Halide-Containing Water. *Environ. Sci. Technol.* **2023,** *57* (47), 18898-18908.
